# Supplementation with cartilage hydrolysate and rosehip peel extract in physically active adults: main outcomes and exploratory findings of a randomized, placebo-controlled study on knee discomfort

**DOI:** 10.3389/fspor.2026.1873793

**Published:** 2026-10-23

**Authors:** Lionel Noah, Cynthia G. Suarez, Arnaud Jacquot, Ralf Spreemann, Christine Juhel, Florian Rigaudier, Etienne Guillocheau, Séverine Méance

**Affiliations:** 1 Symrise (Diana SAS), Saint-Nolff, France; 2 Martin Bauer Nutraceuticals S.r.l., Rivalta di Torino, Italy; 3 CEN, Dijon, France

**Keywords:** cartilage hydrolysate, chondroitin sulfate, collagen, hyaluronic acid, joint discomfort, knee, menopause, Rosa canina

## Abstract

**Background and objectives:**

Joint discomfort and pain during physical activity are frequent conditions in healthy adults, which can lead to mobility limitations, with multiple consequences on health and quality of life. This study aimed to evaluate the effects of a food supplement which combines cartilage hydrolysate and a rosehip extract on knee pain and discomfort in physically active healthy adults, examining subgroups according to sex and menopausal status.

**Methods:**

In this randomized, double-blind, placebo-controlled trial, 138 participants received either 4 capsules/day of Chondractiv™ Move (CM, containing 1200 mg/day of hydrolyzed chicken cartilage and 380 mg/day of Rosa canina extract) or a placebo (maltodextrin) for 8 weeks. Activity-related knee pain was assessed on a 0-10 eVAS following a standardized single-leg step-down test (SLSD) and analyzed via an ANCOVA. Activity-related knee pain experienced during or after daily activities (0-100 eVAS), daily life pain (ICOAP questionnaire), knee function (KOOS questionnaire), quality of life (SF-12 questionnaire) and range of motion were also assessed throughout the intervention period.

**Results:**

Participants (39.9% men, 39.1% pre-menopausal women [PreMW] and 21.0% post-menopausal women [PostMW]) with a mean age of 46.4 years and a mean BMI of 24.4 kg/m^2^, practiced 5.6 hours of exercise per week on average. Knee pain after SLSD significantly decreased (*p* < 0.0001) during the intervention in both arms in the entire population (CM: −2.2; placebo: −2.4), but with no significant difference between arms (delta −0.2 [95%CI −1.1; 0.7], *p* > 0.05). However, exploratory analyses in PostMW showed that the change from baseline tended to be greater with CM versus placebo (−3.4 versus −1.2; delta 2.2 [95%CI −0.2;4.7], *p* = 0.0712). Further exploration indicated a benefit of CM over placebo on knee pain experienced during daily activities (entire cohort; *p* = 0.0255) and after activity (PostMW only; *p* = 0.0421), and in the Sport and recreation dimension of the KOOS (PostMW; *p* = 0.0381). Quality of life and range of motion evolved favorably, but with no between-arm difference.

**Conclusions:**

After eight weeks of supplementation, CM did not demonstrate superiority over placebo for the primary endpoint (pain after SLSD) but exploratory analyses indicated clinically meaningful improvements in postmenopausal women, particularly. These findings require confirmation in further large-scale studies.

**Clinical trial registration:**

ClinicalTrials.gov, identifier [NCT06714851].

## 1 Introduction

Joint discomfort, manifesting as knee pain (or gonalgia), is a common issue not only in elite athletes but also in physically active individuals engaging repeatedly in exercise or sports over time. Even in the absence of a radiographically-confirmed pathology such as osteoarthritis (OA), many healthy persons report transient pain, stiffness or reduced mobility in the knee following periods of repetitive loading. Statistics on knee discomfort, with no overt OA, are not available, but prevalence is surely much higher than that of OA, which is a common condition in the adult population. Indeed, large scale studies report a global prevalence of 16% in people over 15 years of age, and 23% in those over 40 years of age (1, 2). Prevalence increases almost linearly after age 40, with the highest rates in those over 70. Women are more at risk than men, especially after menopause, with studies reporting rates from 41% to nearly 60% (3).

Such functional joint discomfort may reflect subclinical alterations: under repeated mechanical stress, the normal equilibrium between cartilage matrix synthesis and degradation can be temporarily perturbed, leading to micro-damage, transient inflammation, or catabolic processes in cartilage and peri-articular tissues. Over time, repeated episodes of overload and insufficient recovery could in theory contribute to long-term cartilage degeneration, even before overt structural OA becomes detectable (4).

Nevertheless, lack of exercise or joint inactivity can worsen the issue, making the cartilage even more prone to damage. Regular (moderate) physical activity, beyond its well-known use as the primary therapeutic approach against the health impacts of a sedentary lifestyle, is also recommended in the prevention, as well as being the first-line treatment for OA, supported by strong evidence and expert consensus (5).

Given these concerns, strategies to support physical activity and maintain normal joint function, notably in sensitive populations like menopausal women, are of particular importance. Dietary supplementation, combined with physical exercise, has attracted interest. Data from the literature indicate that collagen-derived supplements may reduce joint pain or discomfort and improve function, in physically active healthy individuals with non-OA joint discomfort (6–11), after 12 to 24 weeks of oral intake. However, the benefit of collagen-based supplements is not always retrieved, implying that the supplement composition, dose and duration are critical to potential efficacy (12).

In addition to collagen peptides, the presence of hyaluronic acid (HA) and chondroitin sulfate (CS) in cartilage hydrolysate is theorized to support joint lubrication, viscoelastic properties of synovial fluid, and to exert chondroprotective or anti-inflammatory effects. Preclinical data provides mechanistic plausibility that orally ingested cartilage-derived molecules might exert systemic and joint-protective effects. Indeed, the oral administration of chicken cartilage bioactives (CS, type II collagen peptides) slowed OA development in rats, reduced serum and synovial inflammatory mediators, and preserved cartilage morphology, with modulatory effects on gut microbiota (13). Clinical data further confirm the role of collagen, CS and HA as sources of precursor molecules that potentially support extracellular matrix homeostasis, modulate local inflammation, or influence synovial fluid properties (6, 14).

Moreover, some scientific literature on well-designed controlled trials indicates that extracts from a subspecies of Rosa canina can reduce pain and improve joint function and mobility, in patients with or without advanced OA (15, 16). Mechanistic investigations further support rosehip's potential: *in vitro* experiments found that active constituents of rosehip reduce inflammatory cytokine production in peripheral blood leukocytes, and decrease expression of matrix-degrading enzymes in IL-1*β*-treated chondrocytes — changes consistent with anti-inflammatory and chondroprotective actions (17). In patients with OA, a proof-of-concept study suggested that the patented combination of rosehip peel extract (17) and collagen hydrolysate could improve knee function (18).

Altogether, these data provide a scientifically reasonable rationale for a mixed supplement, combining rosehip extract with cartilage-derived bioactives. This combination is intended to provide a two-pronged approach: (1) supply of structural components/precursors (collagen peptides, CS, HA) for matrix maintenance and synovial fluid support from hydrolyzed cartilage, and (2) anti-inflammatory and chondroprotective action from rosehip — possibly synergistic in the context of mechanical stress or activity-related discomfort, even before overt cartilage degeneration.

In this context, the present study aims to assess the effect of Chondractiv™ Move (CM), a food supplement combining rosehip extract and hydrolyzed chicken cartilage as a source of collagen II, CS, and HA on joint discomfort induced by physical activity in adults exposed to repeated joint load (athletes, recreational sport practitioners) — but without diagnosed OA. Particular attention was paid to subgroups based on sex and menopausal status.

## 2 Materials and methods

### 2.1 Study design and participants

A randomized, double-blind, single site, placebo-controlled, parallel-arm trial was designed to investigate the effects of CM in adult participants and was performed between November 2024 and May 2025. Participants were recruited in the community by an investigational center located in Dijon (France).

To be eligible to the study, male and female volunteers had to fulfil the following criteria: 1) aged 30 65 years; 2) BMI of 18.5-30 kg/m^2^; 3) practicing joint bearing sports for at least 3 hours or twice per week and experiencing knee discomfort for at least 2 months; 4) rating pain as ≥5 on a 0-10 numeric scale after a standardized single-leg step-down test (SLSD), 5) having no other local injuries or diseases that requires analgesic treatments, and no history of intra-articular injections or infiltrations in the last 3 months. Participant eligibility was assessed during the inclusion visit on day 0 (D0).

This study was approved by an Independent Ethics Committee named *Comité de Protection des Personnes Ile de France X* (Aulnay-sous-Bois, France) and the French Health Authority (ANSM) was notified of the study approval. A signed informed consent form (Appendix A in Supplementary material) was obtained from all participants involved in the study before inclusion. The trial protocol was registered at ClinicalTrials.gov in November 2024 (https://clinicaltrials.gov/study/NCT06714851).

### 2.2 Randomization and blinding

After inclusion on D0, each subject was randomly assigned to either the active product Chondractiv™ Move (CM) or placebo. A block randomization list was generated using the SAS software version 9.4 (SAS Institute Inc., Cary, NC, USA). Product allocation was implemented using a minimization randomization method within the Ennov Clinical® software version 8.2 (Ennov, Paris, France), to minimize imbalance between the two treatment arms according to the predefined strata: men, premenopausal women (PreMW), and postmenopausal women (PostMW). Participation in an ancillary study was offered to the first subjects included, on a voluntary basis, using the same randomization method. The investigator assigned the product through an interactive web response system (Ennov RTSM). Investigators and participants were blinded to intervention assignments until the study was completed.

### 2.3 Intervention and procedures

From inclusion, all participants underwent different medical examinations and data collection during visits at the clinical investigation center [on D0 (V0), D28 (V1) and D56 (V2)] including blood sampling (V0 and V2 only), functional tests and self-assessment on electronic questionnaires to collect patient-reported outcomes (PROs): time and type of every physical activity, related pain and rescue medication if any, and 3-day dietary surveys during the first week of supplementation (W1), the week before V1 (W4) and the week before V2 (W8). They were also asked to complete questionnaires at home on D14 and D42, using a secured smartphone application (ePRO Nurstrial®, CEN, Dijon, France) (19, 20). In the ancillary subgroup, participants were required to continue supplementation for up to 12 weeks, to complete self-assessments, including questionnaires on D70 and the 3-day dietary survey during the last week of supplementation, and to attend an additional visit (V3) on D84. The study design is summarized in Figure 1.

**Figure 1:**
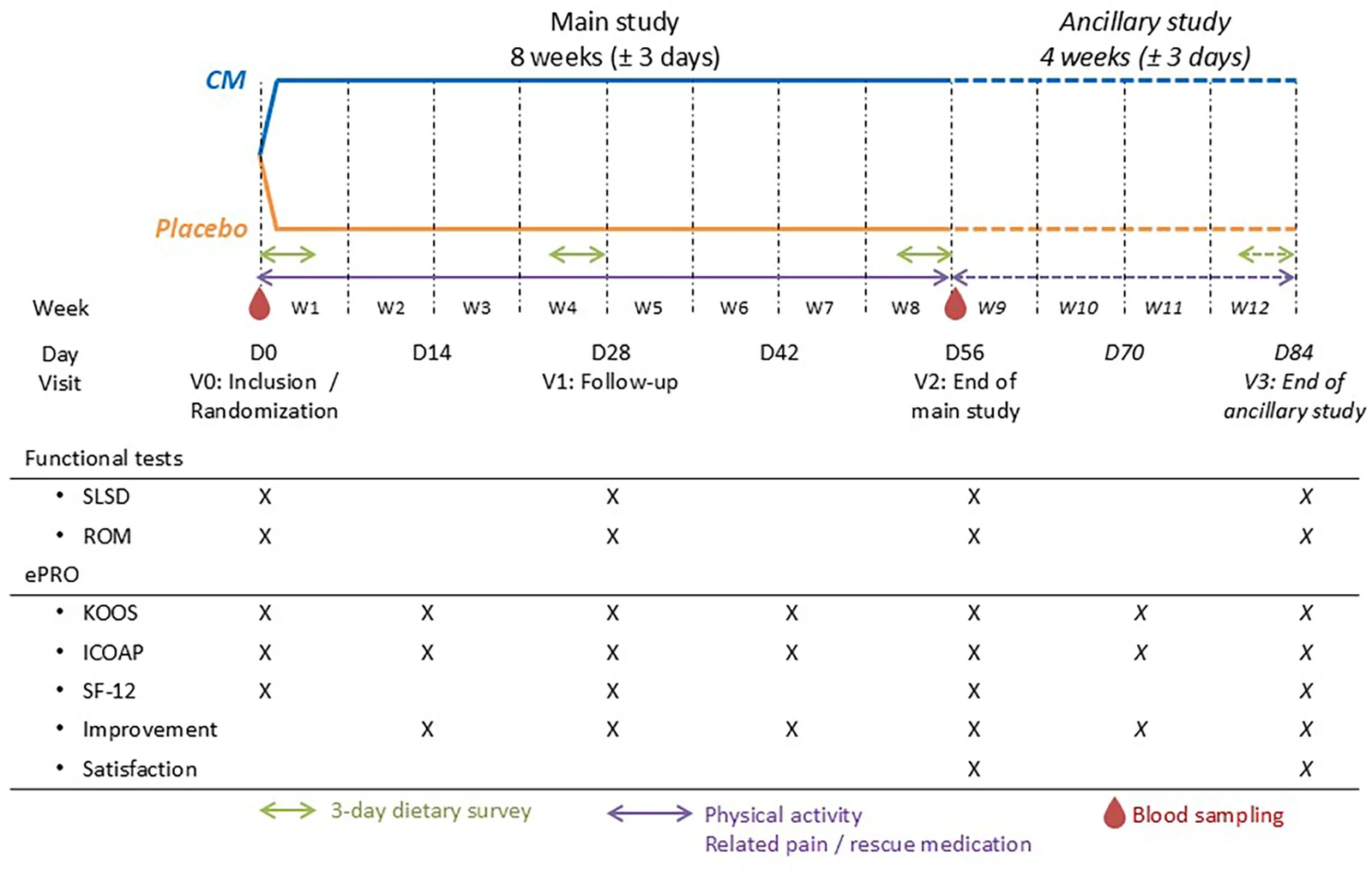
Study design. CM, Chondractiv™ Move; SLSD, single-leg step-down test; ROM, range of motion; e-PRO, electronic patient reported outcomes; KOOS, Knee injury and Osteoarthristis Outcome Score; ICOAP, Intermittent and Constant Osteoarthritis Pain.

Participants were instructed to consume orally four capsules per day of CM or placebo, either with a single meal (preferably breakfast), or spread out over two meals (preferably breakfast and dinner). Both investigational products were provided by Symrise (Saint-Nolff, France). The active product—marketed under the name of Chondractiv™ Move—was a combination of 300 mg of standardized hydrolyzed chicken cartilage (including at least 75 mg of collagen Type II, 45 mg of CS and 3.6 mg of HA) and the patented rosehip extract Agilirose® (95 mg of rosa canina extract, including 6.65 mg of total polyphenols). Chondractiv Move™ nutritional values (per 100 g): Energy 297 kcal, Total Fat 1.7 g (0.4 g saturated fat), Total Carbohydrate 18.3 g (4.7 g sugars), Total Proteins 52 g, Fibers 3.7 g, Sodium 914 mg, Calcium 79 mg, Iron 0.2 mg, Potassium 1017 mg, Ash 6.5 g

The placebo was maltodextrin (per 100 g): Energy 386 kcal, Total Fat 0 g, Total Carbohydrate 95 g (7 g sugars), Total Proteins 0 g, Fibers 0 g, Salt: 200 mg. Both capsules contained 450 mg of products and were similar in color and appearance. An unblinded person (not involved in data collection) was dedicated to products’ preparation and delivery to the investigator. The blinding process was organized in order to ensure that neither the subjects nor the investigating team (collecting the data) could distinguish between the tested products.

Participants were requested to maintain their regular diet and sport practice during the intervention period and were instructed to refrain from taking corticoids and non-steroid anti-inflammatory drugs (NSAIDs), as well as any dietary supplements or herbal medicines that could interfere with joint, tendon or bone metabolism. They were allowed to take their usual painkillers (preferably: paracetamol) during the study, if required, except during the 6 hours preceding the single-leg-step-down (SLSD) tests. Otherwise, all concomitant treatments deemed necessary by the subject's clinical condition were authorized for the duration of the study.

### 2.4 Endpoints

The primary efficacy endpoint was the change from D0 to D56 in knee pain assessed on a 0-10 electronic visual analog scale (eVAS) after a standardized SLSD test. The test involved climbing on and off a height-adjustable platform with the foot of the tested leg staying on the platform, while the opposite leg's foot brushed the ground then returned up to the platform to full knee extension. The up and down movement was repeated every 3 s, corresponding to a maximum of 200 steps within 10 min (21). The test was standardized for each participant, with the platform height equal to the shank length divided by 2.3. The SLSD test at D0 was used to standardize the number of repetitions: number necessary to reach a pain intensity rated at least 5 on a 0-10 numeric scale. These parameters (height and number of repetitions) were reused at each visit to assess pain on the 0-10 eVAS. The change in knee pain intensity from D0 to D28, from to D84 (ancillary subgroup), as well as in the other subgroups (men, women, PreMW and PostMW) from D0 to D56, were secondary endpoints.

Pain [during and within 2 hours after each session of sport practice (training or competition)] was also evaluated using a 0-100 eVAS on the e-PRO application, which computed the average weekly pain intensity during the entire study period. Several validated questionnaires were self-completed on the e-PRO application to assess the overall physical function [Knee injury and Osteoarthristis Outcome Score -KOOS (22, 23)], quality of life [SF-12 (24)], and experience of pain [Intermittent and Constant Osteoarthritis Pain—ICOAP (25)]. Knee range of motion (maximal flexion and extension) were measured by the investigator using a long-arm goniometer including a clamping screw to secure the measurement (GIMA 360° GONIOMETER 14”, Gima SpA, Gessate, Italy). Impression of improvement of joint function/comfort during physical activity or daily activities were self-rated on an ad-hoc 7-point Likert scale (from 1: Improved, to 7: Worsened) (26). A blood sample was taken from each volunteer at D0 and D56, after completion of the different tests, meaning approximately 30-60 min post SLSD. Subjects were instructed to consume their daily dose at breakfast, before the visit. The cartilage oligomeric matrix protein (COMP) and the type IIA collagen N-propeptide (PIIANP) were then measured in the serum as biomarkers of cartilage metabolism (27) (exploratory endpoints), using an indirect ELISA assays (Human COMP ELK1678 Kit and Human PIIANP ELK8809 Kit—ELK Biotechnology, Sugar Land, TX, USA).

Finally, adverse events were recorded throughout the study and analyzed as regards their causality link with the research or the investigational product, their intensity and seriousness, and the system or organ in question.

### 2.5 Statistical analysis

An evaluable sample of 64 participants per arm was expected to provide a statistical power of 80% to detect a difference between active and placebo arms’ effects of 0.5 with standard error of 1.0 on the change in 0-10 pain eVAS. These conservative estimates were based on previous publications (8, 21). Considering a dropout rate of 10%, 72 participants were expected to be randomized in each arm (144 in total). For the ancillary study, 25 participants were expected per arm. The subgroup analyses according to sex (men/women) and menopausal status (premenopausal/postmenopausal), as well as the ancillary study, were prespecified but considered exploratory, and were therefore not separately powered.

For all statistical tests, the 0.05 level of significance has been used to declare a statistically significant effect. The 0.1 level was also considered to discuss trends. All statistical analyses were performed using SAS version 9.4 on the full analysis set (FAS, randomized subjects for whom the primary endpoint was assessable) and the per-protocol set (PPS; participants from the FAS without any major protocol deviation). The results presented in this article primarily concern the FAS, analyses on the PPS were supportive. The missing data (only 0.2% of the e-VAS data in the FAS population) has not been replaced. However, to mitigate the possible impact (i.e., a week without doing exercise), the analysis was slightly modified from what was planned in the protocol, before unblinding: the average intensity of pain during/after exercise sessions (eVAS-E and e-VAS-A) was calculated on a 2-week basis, instead of 1-week.

The primary endpoint was the change from baseline to D56 of the knee pain score (0-10 eVAS) induced by the SLSD test. It was compared between arms using a two-way ANCOVA Time x Product adjusted on the baseline value, in a superiority scenario. Comparisons between arms at each visit were performed using Student unpaired T-tests, while comparisons between visits within each arm were performed using Student paired T-tests. The effect size was assessed using Cohen's d coefficient. Other endpoints were compared between treatment arms over time using an ANOVA on repeated measures (continuous variables), or a Wilcoxon rank-sum test (discrete variables). The subgroup analyses performed on all these endpoints were prespecified. However, given the multiplicity of secondary endpoints, time points and subgroup analyses, no formal adjustment for multiple testing was applied, as the primary hypothesis was restricted to the prespecified primary endpoint. Accordingly, secondary and subgroup analyses were interpreted cautiously, based also on the magnitude, direction and consistency of observed effects, rather than on nominal statistical significance alone.

## 3 Results

### 3.1 Characteristics of participants

As presented in Figure 2, 178 participants were screened in this study, out of whom 144 were randomized based on the selection criteria. The subjects were randomly assigned to the CM arm (*n* = 72) or the placebo arm (*n* = 72). One of the participants from the CM arm did not ingest any product and was therefore excluded from the Safety set. Overall, 6 participants could not be assessed for the primary endpoint and were excluded from the FAS (*n* = 138); 19 participants presented major deviations to the protocol and were excluded from the PPS (*n* = 119). Fifty volunteers (25 in each arm) participated in the ancillary study; they represented 49 participants in the ancillary Safety set, 47 in the ancillary FAS and 39 in the ancillary PPS.

**Figure 2:**
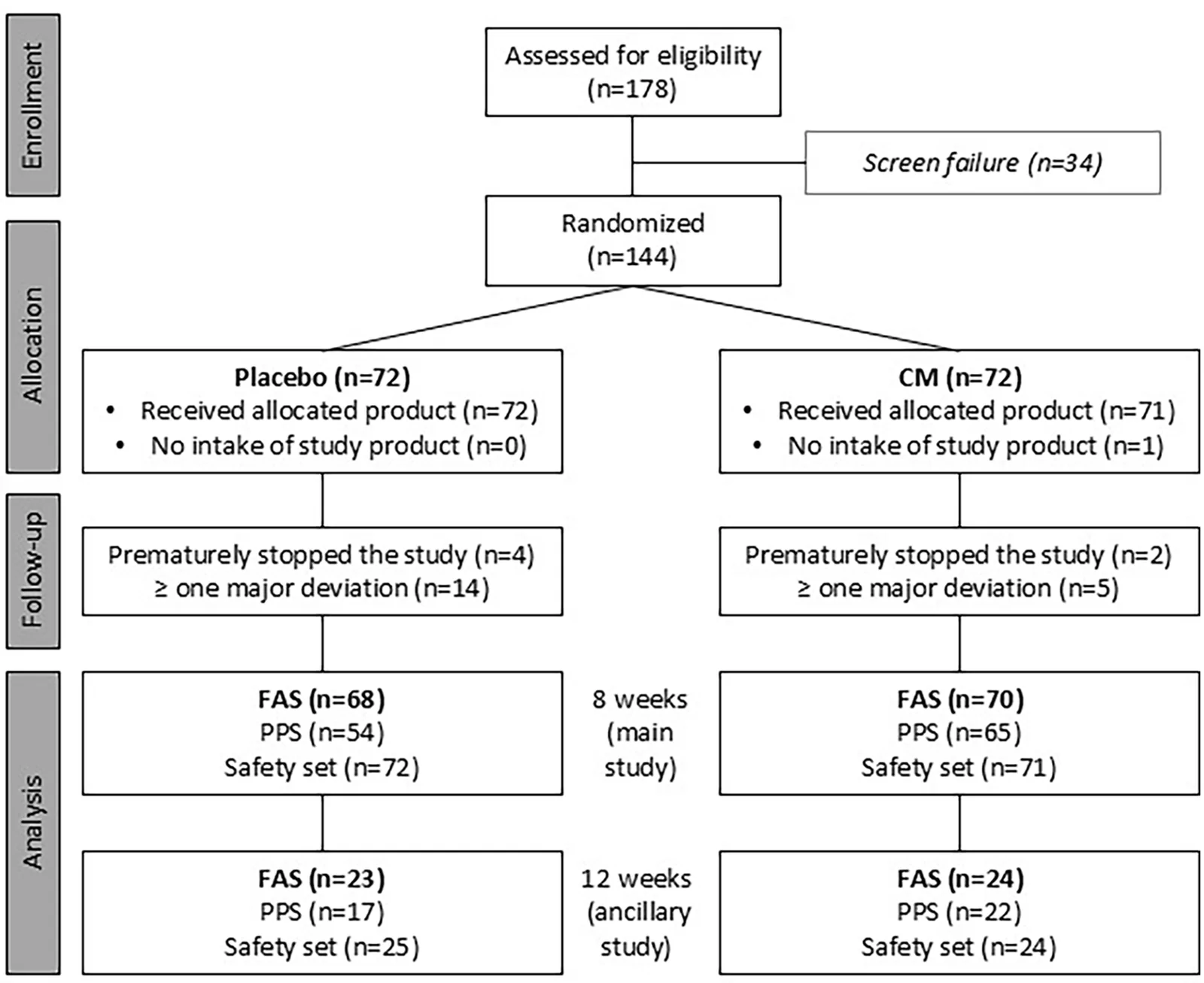
Study flow chart. FAS, full analysis set; PPS, per protocol set; CM, Chondractiv™ Move.

Main baseline parameters of the FAS are presented in Table 1. Data related to knee discomfort, quality of life and biomarkers are presented in (Supplementary Tables S1, S2). Data were quite similar between randomization arms, and no meaningful difference was observed between the FAS and the PPS. On average, PostMW were older than men, who were slightly older than PreMW. The body mass index was comparable in the two subgroups of women, and a little lower in women than in men. Sex also differed by the mean weekly exercise time and the duration of knee pain (higher in men), while functional and quality of life scores were similar when comparing men, PreMW and PostMW (Table 1 and Supplementary Tables S1, S2). Of note, PostMW experienced more intermittent and constant pain at baseline in the placebo arm than in the CM arm, as per the ICOAP. In addition, lower levels of PIIANP were observed at baseline in PostMW of the CM arm, as compared to other subgroups, but the inter-individual variability was high for this biomarker.

**Table 1:** Baseline characteristics per study arm and subgroup in the FAS.

Variable	Placebo All (*n* = 68)	Placebo Men (*n* = 26)	Placebo PreMW (*n* = 28)	Placebo PostMW (*n* = 14)	CM All (*n* = 70)	CM Men (*n* = 29)	CM PreMW (*n* = 26)	CM PostMW (*n* = 15)
Sex								
- Male, n (%)	26 (38.2)	26 (100)	-	-	29 (41.4)	29 (100)	-	-
- Female, n (%)	42 (61.8)	-			41 (58.6)	-	*-*	*-*
o *PreMW*	*28 (41.2)*	-	*28 (100)*	*-*	*26 (37.1)*	-	*26 (100)*	*-*
o *PostMW*	*14 (20.6)*	-	*-*	*14 (100)*	*15 (21.4)*	-	*-*	*15 (100)*
Age (years)								
Mean (SD)	45.0 (9.8)	42.8 (8.8)	40.6 (6.7)	57.8 (4.5)	47.7 (10.4)	48.3 (10.3)	40.5 (6.0)	58.7 (5.9)
Median (Q1; Q3)	45.0 (35.5; 52.0)	43.0 (35.0; 48.0)	42.0 (34.0; 45.0)	59.0 (52.0; 61.0)	46.0 (40.0; 59.0)	48.0 (40.0; 56.0)	40.5 (36.0; 45.0)	60.0 (55.0; 63.0)
BMI (kg/m^2^)								
Mean (SD)	24.3 (2.7)	25.1 (2.3)	23.8 (2.7)	23.9 (2.8)	24.5 (2.9)	25.4 (2.7)	23.7 (2.9)	24.0 (2.9)
Median (Q1; Q3)	24.4 (22.5; 26.5)	25.0 (23.2; 27.5)	23.7 (22.0; 25.8)	24.4 (21.7; 26.0)	24.3 (22.7; 26.7)	25.3 (23.2; 27.4)	23.7 (21.4; 25.3)	24.1 (22.5; 25.6)
Weekly exercise time (h)								
Mean (SD)	5.4 (1.9)	6.1 (2.2)	4.8 (1.6)	5.3 (1.7)	5.8 (3.3)	6.6 (4.0)	5.4 (3.1)	4.9 (1.4)
Median (Q1; Q3)	5.0 (4.0; 6.3)	5.5 (4.0; 8.0)	4.0 (4.0; 5.8)	5.1 (4.0; 6.0)	4.8 (3.8; 7.0)	5.0 (3.8; 7.0)	4.0 (3.8; 6.0)	4.5 (3.8; 6.0)
Duration of knee pain (months)								
Mean (SD)	39.0 (45.0)	49.5 (43.6)	31.1 (35.4)	35.4 (61.6)	41.8 (53.6)	46.4 −50.2)	29.3 (29.8)	54.5 (83.8)
Median (Q1; Q3)	24.0 (6.0; 54.0)	36.0 (12.0; 96.0)	21.0 (7.5; 36.0)	12.0 (6.0; 36.0)	24.0 (12.0; 48.0)	30.0 (24.0; 48.0)	18.0 (8.0; 48.0)	20.0 (12.0; 48.0)

CM, Chondractiv™ Move; PreMW, premenopausal women; PostMW, postmenopausal women.

The type and number of weekly activities reported at baseline differed between sex (Figure 3). Running was the most practiced sport, either by men or women, followed by fitness. As compared to men, women more often practiced swimming, active walking (including golf), fitness and indoor gymnastic activities (including yoga, Pilates, stretching). Men participated in team sports more than women.

**Figure 3:**
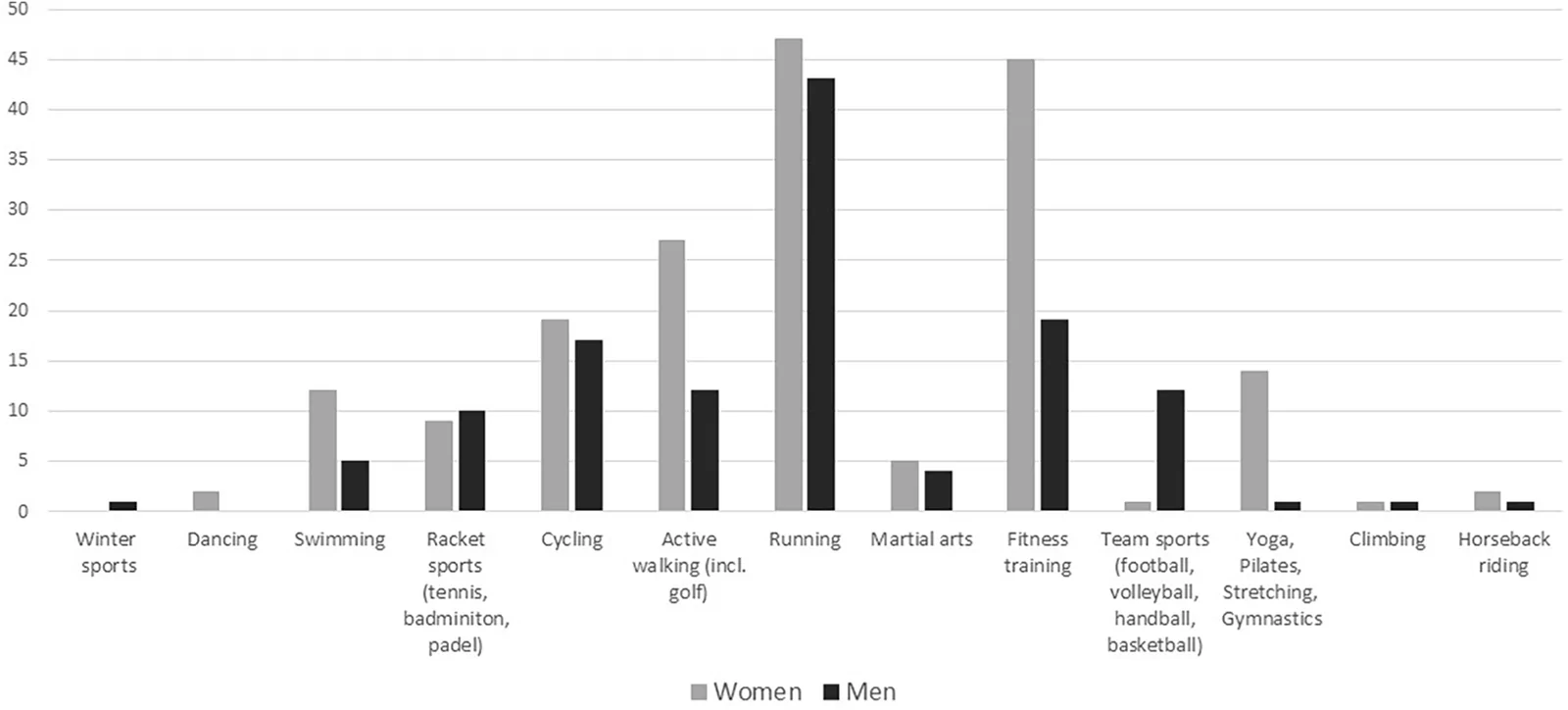
Weekly activities reported at inclusion. Cumulative number of sport sessions reported at inclusion during the previous week by women and men.

Compliance was high in both treatment arms and at all visits (mean ratio of effective/theoretical sachets intake > 95%).

### 3.2 Efficacy outcomes

#### 3.2.1 Stress-induced knee pain decreased during the supplementation, with no benefit of CM over placebo, but a strong tendency in the subgroup of post-menopausal women

Knee pain intensity after the standardized SLSD stress test, self-assessed on the 0-10 eVAS in the entire population (FAS), significantly decreased between baseline and 8 weeks of treatment (D56) in the placebo arm (−2.4; *p* < 0.0001) and in the CM arm (−2.2; *p* < 0.0001, Figure 4) (estimates adjusted on baseline values)**.** However, no significant difference between arms were observed for these changes (delta −0.2 [95%CI −1.1; 0.7], *p* > 0.05). Similar results were obtained when considering subgroups of men, women and PreMW. Conversely, a trend towards a treatment effect was observed in the subgroup of PostMW, reduction of knee pain from D0 to D56 tending to be different between groups (−1.2 versus –3.4; delta 2.2 [95%CI −0.2; 4.7], *p* = 0.0712). A trend was observed (*p* = 0.0791) when testing the intervention * menopausal status interaction. Interestingly, the reduction at D56 from D0 in the placebo arm was not statistically significant, suggesting a lower placebo effect in this sub-population, while the reduction in the CM arm appeared larger than in the other subgroups.

**Figure 4:**
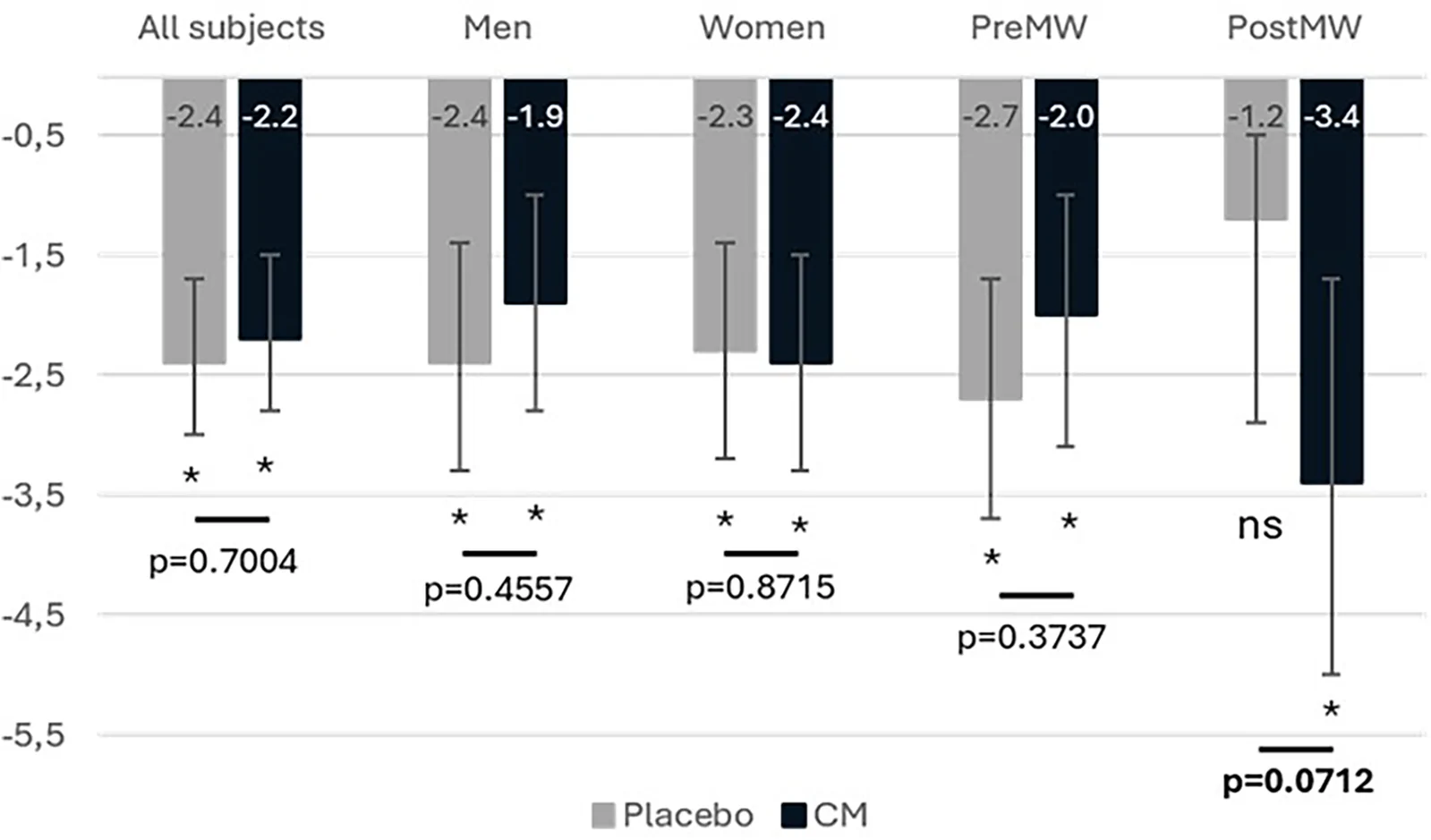
Evolution of knee pain induced by the knee stress test (SLSD), per treatment arm and subgroups (FAS). Values are self-rating of pain on a 0-10 electronic visual analog scale after a standardized knee stress test (SLSD). Data are expressed as estimated means for change from D0 to D56 (*Δ*), adjusted on the baseline value, with its 95% confidence interval. Intra-arm change versus baseline (Student paired t-test): statistically significant (*, *p* < 0.01); non-significant (ns). P-values are for inter-arm difference (CM versus placebo) as per the ANCOVA (arm x time interaction) with an adjustment on baseline. Number of subjects analyzed (placebo/CM arm): All subjects (68/70), Men (26/29), Women (42/41), PreMW (28/26), PostMW (14/15). CMM, Chondractiv™ Move; PreMW, premenopausal women; PostMW, postmenopausal women.

A larger decrease in pain was observed in the ancillary subgroup, with participants continuing the supplementation until 12 weeks (D84), but no significant between-arm difference was observed either (−3.1 in both arms; delta 0.0 [95%CI −1.7; 1.6], *p* = 0.9619).

#### 3.2.2 Knee pain experienced during and after sport sessions also decreased during the supplementation, with a benefit of CM over placebo in the entire population, driven by the subgroup of post-menopausal women

The average pain intensity during and after each exercise session over two weeks, self-rated on a 0-100 eVAS, also significantly decreased over time in both arms in the entire cohort. In the exploratory subgroups, the change was not statistically significant in the placebo arm for men during exercise, nor for PostMW either during or after exercise, while it significantly decreased in the CM arm for those two subgroups (Table 2 and Figure 5). When comparing the values throughout the supplementation, CM significantly reduced the average pain felt during exertion in the entire population as compared to the placebo (*p* = 0.0255), although values at each individual time point did not differ between the two groups. An tendency for a better effect of CM was also observed in the subgroup of women (*p* = 0.0622), and in the PostMW (*p* = 0.0568, with a large effect size: Cohen's d = 0.85 and a pain level 36% lower than that of the placebo arm) and PreMW subgroups (*p* = 0.0945). Regarding pain felt after exertion, a benefit of CM over placebo was found in women (although non-statistically significant: *p* = 0.0735), and even more in PostMW (*p* = 0.0421; Cohen's d = 0.99 and a pain level 39% lower than that of the placebo arm), but not in PreMW (*p* > 0.3) nor in the entire cohort (*p* > 0.1) (Table 2 and Figure 5). No significant interaction was found between time * intervention * menopausal status (*p* = 0.2588).

**Table 2:** Evolution of knee pain experienced during (left) and after (right) exercise sessions, per treatment arm and subgroups (FAS).

	Pain during exercise			Pain after exercise		
Subgroups (and period of time considered)	Placebo	CM	*P-Value (Placebo vs CM)*	Placebo	CM	*P-Value (Placebo vs CM)*
**All subjects**	***n*** **=** **67**	***n*** **=** **70**		***n*** **=** **67**	***n*** **=** **70**	
- W1-W2	38.0 (17.4)	41.1 (16.1)	*0.2822 ^(2)^*	42.8 (17.6)	43.6 (16.3)	*0.7989 ^(2)^*
- W3-W4	35.8 (19.8)	35.0 (18.2)	*0.8011 ^(2)^*	38.4 (19.0)	35.9 (19.5)	*0.4404 ^(2)^*
- W5-W6	33.2 (18.6)	29.6 (18.3)	*0.2610 ^(2)^*	34.2 (18.5)	29.8 (19.3)	*0.1745 ^(2)^*
- W7-W8	30.4 (19.2)	30.0 (19.5)	*0.9088 ^(2)^*	31.7 (19.5)	29.4 (20.3)	*0.5109 ^(2)^*
*P-Value (Δ) ^(1)^*	*<0.0001*	*<0.0001*	***0.0255*** *^(2)^*	*<0.0001*	*<0.0001*	*0.1611 ^(2)^*
**Ancillary subgroup**	***n*** **=** **22**	***n*** **=** **24**		***n*** **=** **23**	***n*** **=** **24**	
- W1-W2	39.5 (19.0)	42.9 (12.4)	*0.4728 ^(2)^*	42.1 (20.8)	42.6 (16.9)	*0.9236 ^(2)^*
- W3-W4	36.2 (21.3)	39.5 (17.0)	*0.5584 ^(2)^*	38.3 (20.2)	38.1 (21.2)	*0.9845 ^(2)^*
- W5-W6	32.1 (17.8)	31.5 (16.6)	*0.8994 ^(2)^*	32.4 (17.4)	28.9 (20.1)	*0.5304 ^(2)^*
- W7-W8	28.8 (20.1)	32.2 (18.7)	*0.5639 ^(2)^*	29.8 (20.7)	28.8 (21.0)	*0.8743 ^(2)^*
- W11-W12	27.0 (21.6)	24.3 (18.2)	*0.6559 ^(2)^*	27.6 (24.4)	21.8 (20.9)	*0.3945 ^(2)^*
*P-Value (Δ) ^(1)^*	*0.0001*	*<0.0001*	*0.5835 ^(2)^*	*<0.0001*	*<0.0001*	*0.6725 ^(2)^*
**Men**	***n*** **=** **25**	***n*** **=** **29**		***n*** **=** **25**	***n*** **=** **29**	
- W1-W2	34.6 (17.4)	39.4 (14.6)	*0.2761 ^(2)^*	42.6 (16.9)	43.2 (17.1)	*0.9113 ^(2)^*
- W3-W4	34.8 (20.1)	38.6 (15.0)	*0.4241 ^(2)^*	37.9 (18.7)	40.3 (18.7)	*0.6399 ^(2)^*
- W5-W6	34.0 (18.6)	32.4 (17.7)	*0.7447 ^(2)^*	34.7 (18.5)	33.2 (19.4)	*0.7850 ^(2)^*
- W7-W8	32.6 (18.8)	32.7 (19.4)	*0.9846 ^(2)^*	32.1 (20.0)	32.3 (20.2)	*0.9664 ^(2)^*
*P-Value (Δ) ^(1)^*	*0.8022*	*<0.0001*	*0.1081*	*0.0005*	*<0.0001*	*0.7025 ^(2)^*
**Women**	***n*** **=** **42**	***n*** **=** **41**		***n*** **=** **42**	***n*** **=** **41**	
- W1-W2	40.0 (17.2)	42.3 (17.1)	*0.5519 ^(2)^*	42.9 (18.3)	43.8 (15.9)	*0.8083 ^(2)^*
- W3-W4	36.4 (19.9)	32.4 (20.0)	*0.3619 ^(2)^*	38.7 (19.3)	32.7 (19.7)	*0.1658 ^(2)^*
- W5-W6	32.7 (18.85)	27.7 (18.6)	*0.2260 ^(2)^*	33.9 (18.8)	27.3 (19.0)	*0.1164 ^(2)^*
- W7-W8	29.1 (19.5)	28.1 (19.5)	*0.8221 ^(2)^*	31.4 (19.5)	27.4 (20.4)	*0.3582 ^(2)^*
*P-Value (Δ) ^(1)^*	*<0.0001*	*<0.0001*	***0.0622*** *^(2)^*	*<0.0001*	*<0.0001*	***0.0735*** *^(2)^*

Values are self-rating of pain on a 0-100 eVAS after exercise sessions (training or competition). Data are expressed as observed mean (standard deviation) during each pair of weeks of the supplementation. P values are obtained from: ^(1)^ Student paired t-test; ^(2)^ ANOVA (arm x time interaction). CM, Chondractiv™ Move.

**Figure 5:**
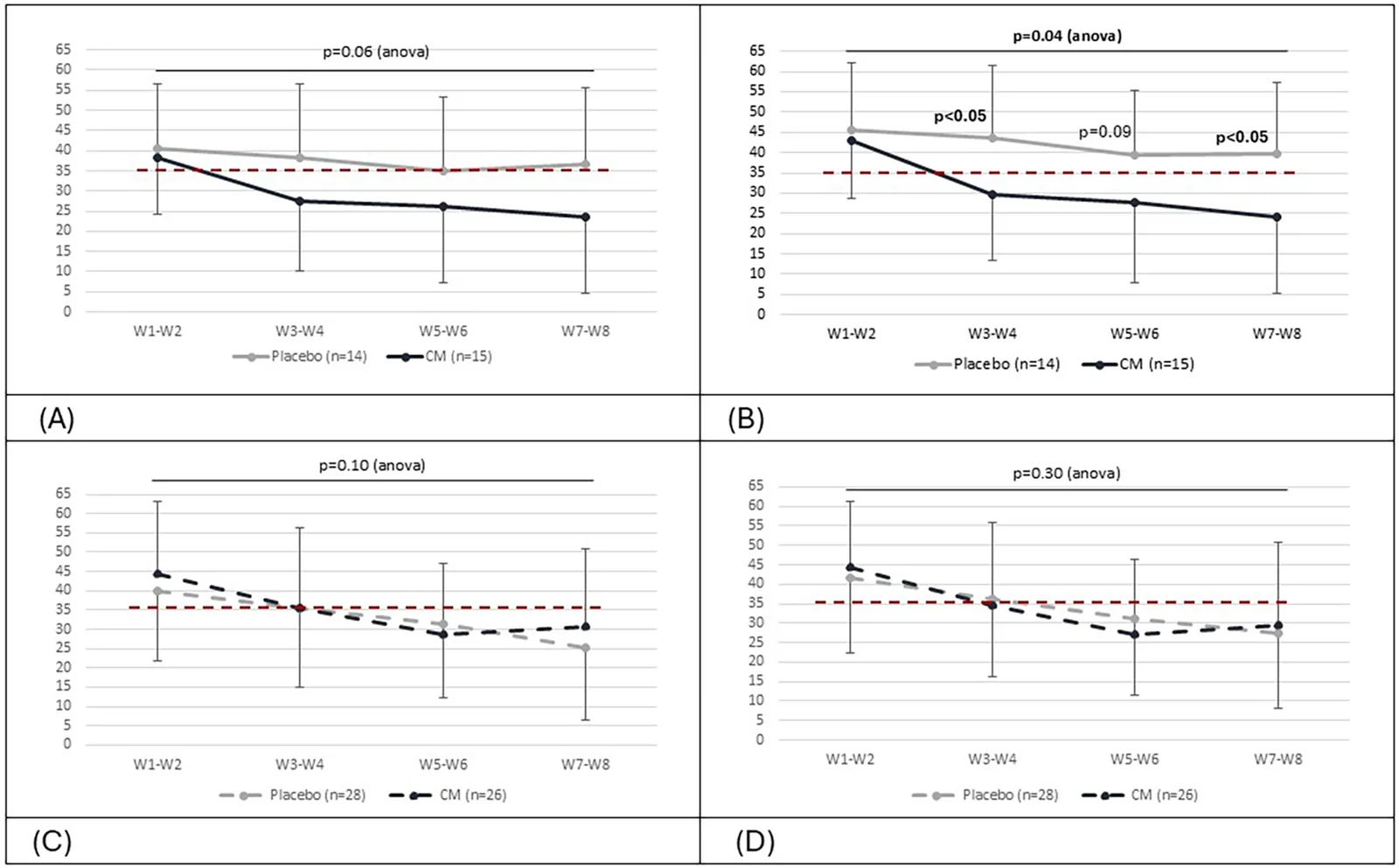
Evolution of knee pain experienced during (left) and after (right) exercise sessions, in subgroups of women (FAS). Values are self-rating of pain on a 0-100 electronic visual analog scale during and after exercise sessions (training or competition) in women subgroups: plain lines represent post-menopausal women during **(A)** or after **(B)** exercise; dotted lines represent pre-menopausal women during **(C)** or after **(D)** exercise. Data are expressed as observed means ± standard deviation over each pair of weeks of supplementation. P values are obtained from ANOVA (arm x time interaction). The red dotted line corresponds to the cut-off point between mild and moderate pain, according to Boonstra. et al. (Boonstra et al. 2014). CM, Chondractiv™ Move.

#### 3.2.3 Intermittent daily life pain decreased under CM

In the entire FAS, intermittent daily life pain, self-assessed using the ICOAP intermittent pain score, decreased in both arms during the supplementation, with trends for a higher effect of CM as compared to that of placebo. Although not significant, the reduction of intermittent pain after 8 weeks of supplementation was 38% greater with CM than with placebo (−14.5 ± 19.6 versus −10.5 ± 16.9; delta 3.9 [95%CI −2.2; 10.1], *p* = 0.2102). Interestingly, the change from baseline to D14 was only significant in the CM arm (−6.0 ± 13.1; *p* = 0.0003) and not in the placebo arm (−2.0 ± 14.5; *p* = 0.2530), although the difference between the two arms did not reach statistical significance, neither on D14 (delta 4.0 [95%CI −0.7; 8.6], *p* = 0.0916) nor on D28 (delta 4.4 [95%CI −0.2; 9.1], *p* = 0.0611) (Figure 6). The exploratory subgroup analysis did not yield significant differences between arms. Regarding the constant pain subscore of the ICOAP, a strong decrease was observed over time in both arms, but with no between-arm difference, regardless of the day or subgroup (in the entire FAS, at D56: −13.5 ± 19.4 for CM versus −12.6 ± 14.5 for placebo; delta 0.9 [95%CI −4.9; 6.6], *p* = 0.7711). Overall, the change in total ICOAP score between D0 and D56 was 22% higher in the CM arm, reaching a clinically meaningful value [i.e.>−13.4, considered as the minimal important change (28)] (−14.0 ± 19.0 for CM versus −11.5 ± 14.8 for placebo; delta 2.5 [95%CI −3.2; 8.3], *p* = 0.3853).

**Figure 6:**
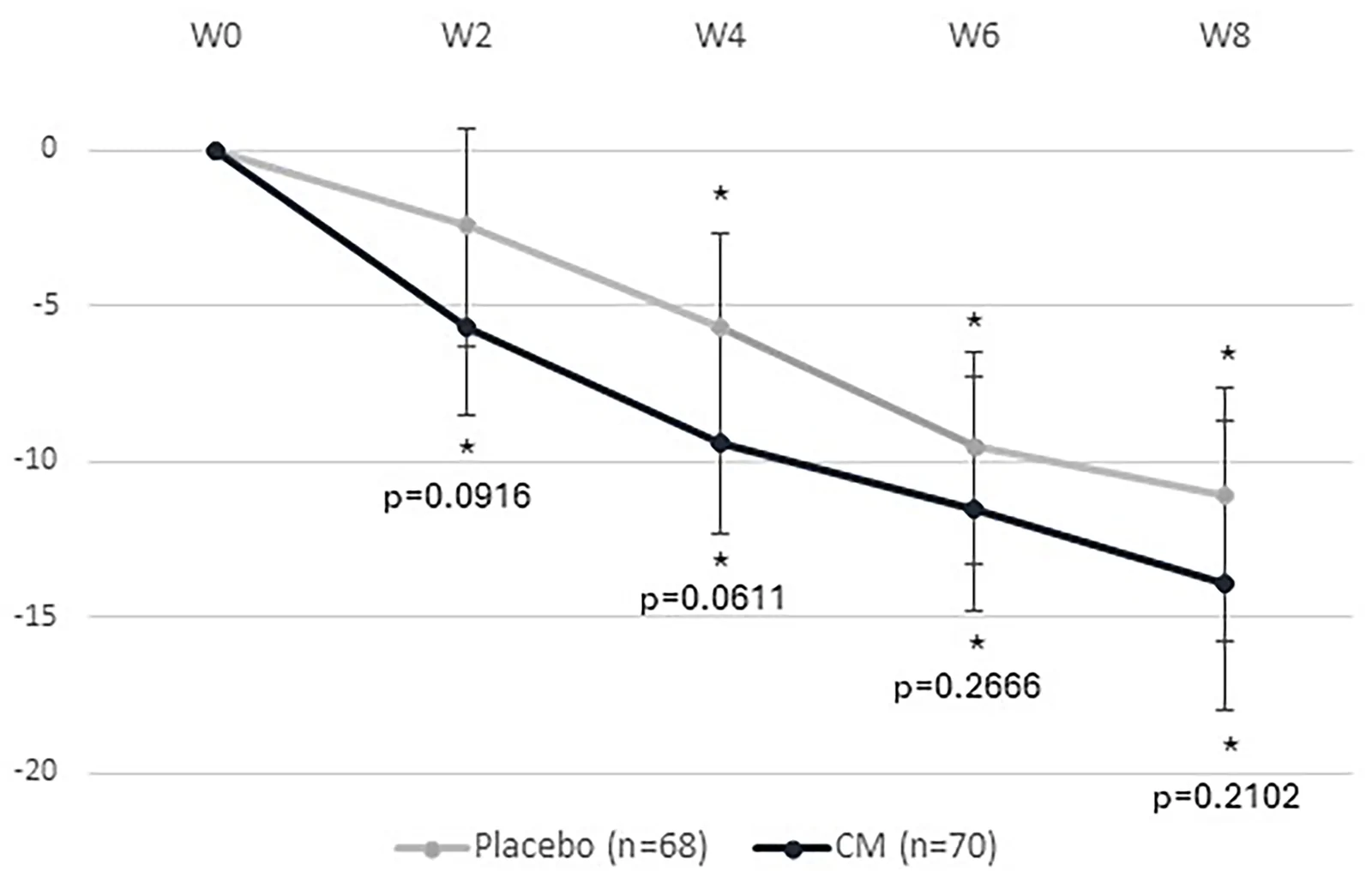
Evolution of intermittent daily life pain (ICOAP) in the entire population (FAS). Data are change from baseline (W0) to end of supplementation (W8) from the Intermittent and Constant Osteoarthritis Pain (ICOAP) questionnaire—intermittent subscore, self-assessed every other week. Data are expressed as estimated means adjusted on the baseline value, with its 95% confidence interval. Intra-arm change versus baseline (Student paired t-test): significant (*, *p* < 0.01); non-significant (ns). P-values are for inter-arm difference (CM versus placebo) as per the ANCOVA (arm x time interaction) with adjustment on baseline. CM, Chondractiv™ Move.

#### 3.2.4 Clinically meaningful differences were observed between CM supplementation and placebo in the improvement of the “sport and recreation” dimension of the knee functional score KOOS in postmenopausal women

Mean changes from baseline in KOOS global score were positive in both arms, indicating an overall improvement in knee function over time. However, the between-arm difference was not statistically significant, either in the entire FAS or in the subgroups (Table 3). In PostMW, the change in KOOS global score was 2.4 times higher under CM than under placebo. Notably, the change from baseline did not reach statistical significance at D56 in the placebo arm (+5.5 ± 12.4; *p* = 0.1220), while this change was significant in the CM group (+13.4 ± 17.6; *p* = 0.0106)(delta −7.9 [95%CI −19.5; 3.8], *p* = 0.1776). When investigating the 5 dimensions of the score (Supplementary Table S1 for baseline values), a significantly higher effect of CM was found versus placebo for the “Sport and recreation” dimension (+3.2 ± 22.8 versus +21.3 ± 21.9; delta −18.1 [95%CI −35.2; −1.1], *p* = 0.0381), with a large effect size (Figure 7; Cohen's d = 0.82). For this dimension, a significant interaction (*p* = 0.0230) was found between intervention * menopausal status. The mean difference between arms exceeded the minimal important difference for two dimensions as determined in the literature (29): “Sport and recreation” (> 17.5) and “Activities of daily living” (>2.5). In addition, the mean change from D0 to D56 reached the minimal important change in three dimensions out of five in the CM arm (but not the placebo arm): “Pain” (>12.4), “Activities of daily living” (>8.4), and “Knee QoL” (>9.8) (29).

**Table 3:** Change from baseline to the end of supplementation for the overall knee functional score (kOOS), per treatment arm and subgroups (FAS).

Subgroups (and timepoints considered)	Placebo	CM	*P-Value (Placebo vs CM)*
**All subjects**	***n*** **=** **68**	***n*** **=** **70**	
- D0	66.8 (12.1)	66.8 (11.8)	*0.9916 ^(2)^*
- D56	77.6 (13.8)	78.6 (13.1)	*0.6700 ^(2)^*
- *Δ* D56-D0	10.8 (11.1)	11.8 (12.9)	*0.6424 ^(3)^*
*P-Value (Δ* D56-D0*) ^(1)^*	*<0.0001*	*<0.0001*	
**Ancillary subgroup**	***n*** **=** **23**	***n*** **=** **24**	
- D0	63.1 (14.9)	68.6 (11.3)	*0.1583 ^(2)^*
- D84	77.2 (15.4)	81.3 (12.4)	*0.3221 ^(2)^*
- *Δ* D84-D0	14.2 (13.6)	12.8 (15.4)	*0.7382 ^(3)^*
*P-Value (Δ D0 to D84) ^(1)^*	*0.0001*	*0.0005*	
**Men**	***n*** **=** **26**	***n*** **=** **29**	
- D0	66.5 (11.0)	64.4 (10.4)	*0.4778 ^(2)^*
- D56	78.6 (13.9)	75.1 (12.4)	*0.3289 ^(2)^*
- *Δ* D56-D0	12.1 (11.3)	10.7 (9.6)	*0.6152 ^(3)^*
*P-Value Δ (D0-D56) ^(1)^*	*P* *<* *0.0001*	*P* *<* *0.001*	
**Women**	***n*** **=** **42**	***n*** **=** **41**	
- D0	67.0 (12.9)	68.5 (12.5)	*0.5868 ^(2)^*
- D56	77.0 (13.8)	81.0 (13.1)	*0.1757 ^(2)^*
- *Δ* D56-D0	10.0 (11.0)	12.6 (14.9)	*0.3809 ^(3)^*
* P-Value (Δ D0-D56) ^(1)^*	*<0.0001*	*<0.0001*	
**Postmenopausal women**	***n*** **=** **14**	***n*** **=** **15**	
- D0	66.8 (15.0)	68.0 (14.5)	*0.8296 ^(2)^*
- D56	72.3 (12.5)	81.4 (15.3)	*0.0932 ^(2)^*
- *Δ* D56-D0	5.5 (12.4)	13.4 (17.6)	*0.1776 ^(3)^*
* P-Value (Δ D0-D56) ^(1)^*	*0.1220*	*0.0106*	
**Premenopausal women**	***n*** **=** **28**	***n*** **=** **26**	
- D0	67.0 (12.0)	68.7 (11.5)	0.5932 *^(2)^*
- D56	79.3 (14.1)	80.8 (12.1)	0.6746 *^(2)^*
- *Δ* D56-D0	12.3 (9.6)	12.1 (13.5)	*0.9475 ^(3)^*
* P-Value (Δ D0-D56) ^(1)^*	*<0.0001*	*<0.0001*	

Values are global score of the Knee injury and Osteoarthritis Outcome Score (KOOS). Data are expressed as observed mean (standard deviation) at D0, D56, D84, and change from D0 (*Δ*). P-values are obtained from: (1) Student paired t-test; (2) Student unpaired t-test; (3) ANOVA (arm x time interaction). CM, Chondractiv™ Move.

**Figure 7:**
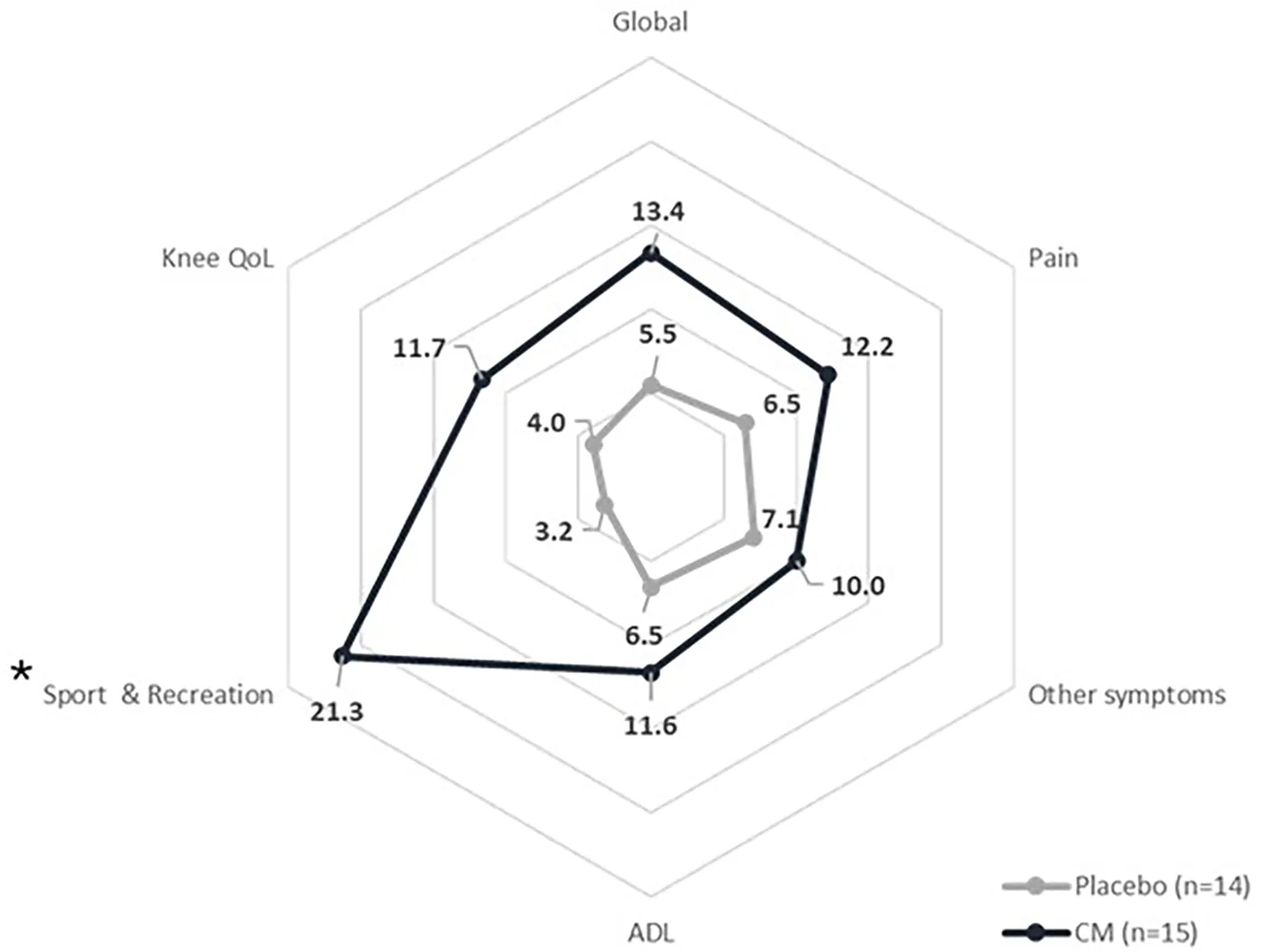
Change from baseline to the end of supplementation in all KOOS dimensions in postmenopausal women (FAS). Values are changes from baseline (D0) to the end of supplementation (D56) of subscores of the Knee injury and Osteoarthritis Outcome Score (KOOS), related to pain, activities of daily living (ADL), sport recreation, knee-related quality of life (QoL), and other symptoms. Data are expressed as observed means. ANOVA applied to the global score and subscores showed a significant difference between arms for the “Sport & recreation” subscore only (*: *p* = 0.0381 for arm x time interaction). CM, Chondractiv™ Move; ADL, activities of daily living.

#### 3.2.5 The range of motion improved after supplementation, with no difference between groups

Active flexion improved significantly versus baseline after 8 weeks of supplementation with CM in the entire FAS (+3.0 ± 8.0; *p* = 0.0023), and even more after 12 weeks in the exploratory ancillary subgroup (+4.4 ± 8.2; *p* = 0.0151), whereas the change was not statistically significant in the placebo arm on D56 (+1.6 ± 8.5; *p* = 0.1259) nor on D84 (+1.8 ± 7.8; *p* = 0.2745, respectively). However, no significant between-arm difference was found, neither at D56 (delta −1.5 [95%CI −4.2; 1.3], *p* = 0.3018) nor at D84 (delta −2.6 [95%CI −7.3; 2.1], *p* = 0.2749).

#### 3.2.6 Improvements in knee function and perceived comfort may be observed following 12 weeks of supplementation

Health-related quality of life (assessed with SF-12) improved over time in both arms, but no significant differences between arms were detected, either in the entire population (Table 4) or in the exploratory subgroups (not shown).

**Table 4:** Change from baseline to the end of supplementation for the scores of health-related quality of life (SF-12), per treatment arm (FAS).

Quality of life components (and timepoints considered)	Placebo	CM	*P-Value (Placebo vs CM)*
**Physical component score**	***n*** **=** **68**	***n*** **=** **70**	
- D0	46.0 (7.7)	44.6 (7.0)	*0.2611 ^(2)^*
- D56	49.6 (6.5)	48.3 (7.0)	*0.2542 ^(2)^*
- *Δ* D56-D0	3.6 (7.5)	3.7 (8.2)	*0.9420 ^(3)^*
*P-Value (Δ* D56-D0*) ^(1)^*	*0.0002*	*0.0003*	
**Mental component score**	***n*** **=** **68**	***n*** **=** **70**	
- D0	48.5 (7.5)	49.9 (8.0)	*0.2918 ^(2)^*
- D56	51.2 (6.7)	53.0 (5.4)	*0.0869 ^(2)^*
- *Δ* D56-D0	2.7 (9.2)	3.0 (7.4)	*0.7822 ^(3)^*
*P-Value (Δ D0 to D56) ^(1)^*	*0.0199*	*0.0010*	

Values are physical and mental component scores of the SF-12 questionnaire. Data are expressed as observed mean (standard deviation) at D0 and D56, and observed mean for change from D0 (*Δ*). P-values are obtained from: (1) Student paired t-test; (2) Student unpaired t-test; (3) ANOVA (arm x time interaction). CM: Chondractiv™ Move.

After 8 weeks of supplementation, the impression of improvement, assessed on a 7-point Likert scale, seemed comparable between CM and placebo, with 32.9% and 26.5% of participants of the FAS (*n* = 138) choosing “improved”, respectively (p-value from Wilcoxon Rank-sum test: *p* = 0.4304). After 12 weeks (ancillary group), a trend for a stronger improvement was observed with CM compared to placebo. Indeed, 50.0% of the subjects had an impression of improvement in the CM arm, versus only 21.7% in the placebo arm; 70.9% participants reported at least a slight impression of improvement, versus 63.2% (*p* = 0.0958).

#### 3.2.7 Changes in biomarkers throughout supplementation suggest an impact on cartilage remodeling

Even high inter-individual variability was observed, the serum COMP concentrations (biomarker of cartilage degradation) significantly increased from baseline to D56 in the CM arm (+10.3 ± 31.1 ng/mL, *p* = 0.0070), while they significantly decreased in the placebo arm (−9.7 ± 33.2 ng/mL, *p* = 0.0189) (delta −20.0 [95%CI −30.8; −9.2], *p* = 0.0004). The change significantly differed between arms after adjustment on the baseline values using an ANCOVA (+5.9 [−0.2; 12.0] versus −5.2 [−11.4; 1.0] ng/mL, delta −11.1 [95%CI −19.9; −2.3], *p* = 0.0135). Conversely, no significant change from baseline, nor between arm difference was found regarding the serum biomarker of cartilage synthesis PIIANP.

### 3.3 Safety

No major side effects were reported, and the intake of CM for 8 or 12 weeks was well tolerated.

## 4 Discussion

The occurrence of joint pain or discomfort must be regarded as a warning signal for any further degradation of joint structures. Rather than reducing physical activity, whose health benefits are well established, limiting joint damage via a dietary-based strategy seems a good option to explore. Literature provides preliminary but incomplete support for the concept that collagen hydrolysate-based, cartilage-derived supplements may reduce joint pain, stiffness, or discomfort in both OA patients and healthy individuals with activity-related joint pain. The biological rationale is plausible, but evidence remains heterogeneous, often limited in duration, and rarely focused on clinical or non-OA populations. If effective, an oral supplement such as CM, containing collagen, CS, HA and rosehip extract as the main bioactives, could help relieve transient activity-induced joint discomfort, delay or prevent long-term cartilage deterioration, and consequently, delay the onset of chronic degenerative disease.

The present study is the first clinical trial to investigate the combined effects of hydrolyzed cartilage (supplying structure precursors for cartilage metabolism) and a rosehip extract (which supplies anti-inflammatory and chondroprotective molecules) on joint discomfort, and that paid particular attention to sex and menopausal status for women. In this population of adults aged 30 to 65 years old experiencing activity-related knee pain, we observed no product effect for the primary endpoint in the entire population, but some interesting outcomes emerged from exploratory analyses on the subgroup of PostMW. Notably, in this subgroup, after 8 weeks of supplementation, we observed strong trends for differences between product arms in the reduction of repeated flexion-induced knee pain (primary endpoint), of knee pain experienced during or after physical activity sessions, and of knee function linked to sport and recreation. In this subgroup, the reduction of pain surrounding physical activities was clinically relevant under CM, since it decreased below the cut-off score between mild and moderate pain (30), from the 3rd week of supplementation onwards. In addition, the relative difference in pain between arms at the end of supplementation was 36% (during exercise) and 39% (after), with large effect size according to Cohen's coefficient.

Food supplements containing collagen hydrolysates of different origins have been studied for their effects on knee pain during exertion in healthy adults. The evidence consistently shows a reduction in activity-related knee pain and improvements in joint function, though data specific to menopausal status are limited. One product made of porcine collagen peptides has been the subject of a few randomized controlled trials, which demonstrated that daily supplementation (typically 5–10 g/day for 12 weeks or longer) significantly reduces knee pain during or after exercise in healthy, physically active adults of both sexes (7–9). A greater reduction in exercise-induced knee pain was observed in the collagen group compared to placebo, as measured by both participant and physician assessments (8). Similar findings were reported in athletes and active adults, with significant improvements in pain during activity but not at rest (7, 9). Collagen hydrolysate supplementation also improved activities of daily living and quality of life scores in healthy adults with knee discomfort (9, 31). A comparable effect was found with a mixture of hydrolyzed collagen, HA, vitamin C, curcuma root extract and resveratrol (32). Most studies included both men and women but did not always report results stratified by sex or menopausal status. However, some trials and reviews note that women, including those in the premenopausal and postmenopausal age ranges, were well represented in study populations (8, 9, 31, 33). In a recent trial, improvements in physical health scores with collagen supplementation were significant in females, suggesting a possible sex-specific benefit (31).

Most studies evaluating combinations of collagen, CS and HA were conducted in patients with established OA. These studies consistently show pain reduction and improved function, but their findings may not directly translate to healthy populations with activity-related joint pain or discomfort (14, 34, 35). We found a few studies that assessed the effects of hydrolyzed chicken cartilage (with collagen hydrolysate, CS and HA as main actives) on joint symptoms, at doses of 2 to 2.5 g/day (compared to 1.2 g/day in our study), but none were conducted in subjects with knee pain during exertion. Results from a randomized, double-blind, placebo-controlled trial conducted in healthy adults (without overt OA) support the idea that an 8-week oral supplementation with hydrolyzed chicken-sternum cartilage may improve non-disease, joint discomfort: a significant reduction of global symptoms and stiffness, but not of pain, was observed. This study recruited adults aged 40 to 65 years old, including 65% of women, but their menopausal status was not reported (6). In an even more advanced situation of confirmed knee or hip OA, another chicken cartilage extract yielded significant reductions in pain, and improvements in function after 10 weeks, when compared with placebo, suggesting that cartilage-derived bioactives can have clinically meaningful effects on symptomatic joint disease in a population of 40 to 70 year-old subjects including 60% of women (36). Still in patients with knee OA, 2 g/day of chicken cartilage hydrolysate for 24 weeks failed to reduce either pain and overall symptoms, although PostMW represented about two thirds of their cohort (37).

Beyond cartilage remodeling, inflammatory reaction is another component of joint pain and discomfort which can be targeted by bioactive molecules originating from botanicals. Various botanical extracts are proposed for the management of joint pain (38, 39), but few studies have investigated their effects in healthy adults engaged in physical exercise (40). Interestingly, rose hip extracts (from seeds and husks, pseudo-fructus) have demonstrated significant effects versus placebo on joint pain and daily inflammation at the dosage of 2.25-5 g daily minimum after 3 months, either in patients with OA (15, 41–44) or in individuals with knee-related walking limitations only (16). On the other hand, Walbroel and Feistel (2009) conducted an *in vitro* study showing that a specific extract (used in the present study) from peels of wild growing rosehip berries reduced inflammation biomarkers (especially IL-8) and cartilage-degrading enzymes MMP1 and MMP3 in IL-1ß stimulated human chondrocytes. While reducing inflammation at the joint level (thus reducing pain perception), this mechanism might contribute to preserving joint structure and function from stress conditions such as overload (45). These results support the idea of combining anti-inflammatory botanical extracts with cartilage precursors, the two potentially acting together to relieve joint pain.

Notably, our results showed an increase of serum COMP levels after the 8-week supplementation with CM, but not with placebo, and no significant change in PIIANP. Serum COMP levels are generally observed as increased during OA progression (46) though conflicting results have also been reported (47). Usually, COMP is found in cartilage, where it contributes to the formation of collagen fibrils, and serum COMP is generally regarded as a biomarker of cartilage turnover during inflammatory processes (48). On the other hand, physical activity is beneficial for the prevention of OA, notably vigorous and moderate recreational activity (49). Serum COMP levels were found increased after a beneficial physical therapy intervention in pre-OA subjects (while cartilage degradation was decreased)—the effect was stronger than in OA subjects (50).

However, COMP may also be regarded as a short-term exercise load “sensor”. Indeed, some studies have shown an elevation of serum COMP levels after walking, running or cycling (51, 52). In the current study, blood samples for biomarker analysis were drawn 30 to 60 min after the SLSD test, so that COMP levels may reflect a short-term activity-related release. In this case, the inter-arm difference could reflect a higher remodeling activity of the cartilage in the CM arm—supplied with structural components—than in the placebo arm, following the SLSD test. We hypothesize that the increase in serum COMP levels induced by CM might indirectly reflect an intra-articular (synovial) anti-inflammatory change, contributing to the overall beneficial effect observed with the supplementation: a decrease in synovial COMP levels, paralleled by a decrease in pro-inflammatory cytokines (53). At the synovial level, it has been proposed that a rise in chronic, low-grade synovial inflammation may precede structural degeneration that accompanies degenerative joint diseases (54–56). Although we lack data from the synovial fluid from our volunteers, this explanation fits with the *in vitro* coculture models which have also identified synovium as the primary tissue source of inflammatory cytokines after load stress injury (57).

Regarding knee motion, there was a trend towards a growing benefit of CM over placebo with time in the whole cohort, with a significant gain of active flexion of +3.0° after 8 weeks of supplementation with CM, and of +4.4° after 12 weeks in the ancillary subgroup, whereas the change remained limited and not significant in the Placebo arm (+1.6° and +1.8°, respectively). Interestingly, in a similar population, Schön et al. (2022) reported a comparable gain in active flexion under undenatured collagen, which tended to increase from 8 to 24 weeks of supplementation (21).

Finally, some results obtained in the ancillary group (supplemented until 12 weeks) tend to indicate that increasing the duration of supplementation with CM may improve the benefits on joint health. Indeed, the rate of subjects reporting an impression of improvement increased from 8 to 12 weeks in the CM arm, while being higher at both time points as compared to the placebo arm. This was also corroborated by the data on active knee flexion, discussed above, which suggest a better gain of motion after a longer supplementation. Our results, obtained in a limited cohort of subjects, provide basis supporting further studies of longer duration.

Our study presents several limitations. We observed much higher than expected reductions of SLSD-induced pain in the two supplementation arms, suggesting a strong placebo effect, which is quite usual in trials on knee OA (58): it might have been underestimated in sample size assumptions. On the other hand, although this has not been reported in previous works (11, 21), we cannot exclude a direct therapeutic effect of the SLSD test itself (5), independent of the supplements tested. This hypothesis is unlikely however, given the low frequency of the visits (monthly), and the subject's profile (practicing joint bearing sports regularly). It should also be noted that the between-arm difference observed in the PostMW subgroup for the primary endpoint relies on a stronger change in the CM arm, as compared to other subgroups, but also on a lower change in the placebo arm. In addition, part of the observed effects concerned secondary endpoints, and were mostly trends, considering p-values <0.1, beyond the statistically significant threshold set at 0.05. Finally, the subgroup analysis involved multiple tests, which led to an inflation of the alpha risk. Therefore, the results should be confirmed in further large-scale studies.

Nevertheless, these results are encouraging and deserve further investigations, especially targeting aging women. PostMW are particularly vulnerable to joint discomfort, with hormonal changes favoring altered joint function (59). This corroborates two major risk factors of OA: female sex and age (3). Physical activity is important for a healthy life, for both mental and physical wellbeing, and even more for healthy aging. It helps preserve joint flexibility, reducing stiffness and discomfort, it stimulates synovial fluid production, nourishing joint cartilage; it also helps control body weight, reducing stress on weight-bearing joints like hips and knees (60, 61). Appropriate food supplementation could be part of a healthy virtuous cycle to support joint health.

## 5 Conclusions

An 8-week supplementation with the dietary supplement CM, containing a cartilage hydrolysate and a rosehip extract, induced a significant decrease from baseline for pain perceived during the knee stress test (SLSD) but demonstrated no statistical superiority versus placebo on this parameter. However, exploratory analyses indicated that PostMW may have benefited the most from the supplementation, with a clinically meaningful improvement versus placebo in pain during/after daily exercise (from the 2nd to 3rd week), and in knee discomfort during sport and recreational activities. Finally, the product was very well tolerated. Further large-scale studies are required to confirm if CM may offer a relevant solution to women over 40 (and even more specifically women after the menopausal transition) who are the most impacted by the overall consequences of physical activity decline after midlife.

## Data Availability

The datasets presented in this article are not readily available because they relate to proprietary findings. The raw data supporting the conclusions of this article, together with the protocol and the statistical analysis plan and statistical code, will be made available upon request. Requests to access the datasets should be directed to lionel.noah@symrise.com.

## References

[1] Cui A Li H Wang D Zhong J Chen Y Lu H. Global, regional prevalence, incidence and risk factors of knee osteoarthritis in population-based studies. eClinicalMed. (2020) 29:100587. 10.1016/j.eclinm.2020.100587 PMC7704420 34505846

[2] Li E Tan J Xu K Pan Y Xu P. Global burden and socioeconomic impact of knee osteoarthritis: a comprehensive analysis. Front Med. (2024) 11. 10.3389/fmed.2024.1323091 PMC11137242 38818397

[3] Xu H Xiao W Ding C Zou J Zhou D Wang J. Global burden of osteoarthritis among postmenopausal women in 204 countries and territories: a systematic analysis for the global burden of disease study 2021. BMJ Glob Health. (2025) 10:e017198. 10.1136/bmjgh-2024-017198 40037907 PMC11891539

[4] Hunter DJ Bierma-Zeinstra S. Osteoarthritis. Lancet. (2019) 393:1745–1759. 10.1016/S0140-6736(19)30417-9 31034380

[5] Daste C Kirren Q Akoum J Lefèvre-Colau M-M Rannou F Nguyen C. Physical activity for osteoarthritis: efficiency and review of recommandations. Joint Bone Spine. (2021) 88:105207. 10.1016/j.jbspin.2021.105207 33962031

[6] Mohammed A He S. A double-blind, randomized, placebo-controlled trial to evaluate the efficacy of a hydrolyzed chicken collagen type II supplement in alleviating joint discomfort. Nutrients. (2021) 13:2454. 10.3390/nu13072454 34371963 PMC8308696

[7] Zdzieblik D Oesser S Gollhofer A König D. Improvement of activity-related knee joint discomfort following supplementation of specific collagen peptides. Appl Physiol Nutr Metab. (2017) 42:588–595. 10.1139/apnm-2016-0390 28177710

[8] Zdzieblik D Brame J Oesser S Gollhofer A König D. The influence of specific bioactive collagen peptides on knee joint discomfort in young physically active adults: a randomized controlled trial. Nutrients. (2021) 13:523. 10.3390/nu13020523 33562729 PMC7915677

[9] Schulze C Schunck M Zdzieblik D Oesser S. Impact of specific bioactive collagen peptides on joint discomforts in the lower extremity during daily activities: a randomized controlled trial. Int J Environ Res Public Health. (2024) 21:687. 10.3390/ijerph21060687 38928934 PMC11203623

[10] Lugo JP Saiyed ZM Lau FC Molina JPL Pakdaman MN Shamie AN. Undenatured type II collagen (UC-II®) for joint support: a randomized, double-blind, placebo-controlled study in healthy volunteers. J Int Soc Sports Nutr. (2013) 10:48. 10.1186/1550-2783-10-48 24153020 PMC4015808

[11] Knaub K Schön C Alt W Durkee S Saiyed Z Juturu V. UC-II® undenatured type II collagen reduces knee joint discomfort and improves mobility in healthy subjects: a randomized, double-blind, placebo-controlled clinical study. J Clin Trials. (2022) 12:492. Available online at: https://www.longdom.org/open-access-pdfs/ucii-undenatured-type-ii-collagen-reduces-knee-joint-discomfort-and-improves-mobility-in-healthy-subjects-a-randomized-d.pdf 10.1089/jicm.2021.0365 PMC9232232 35377244

[12] Yuenyongviwat V Anusitviwat C Tuntarattanapong P Hongnaparak T Iamthanaporn K. Efficacy of combined undenatured type II collagen and hydrolysed collagen supplementation in knee osteoarthritis: a randomised controlled trial. Sci Rep. (2025) 15:32313. 10.1038/s41598-025-17505-0 40897777 PMC12405528

[13] Zhang H Qi L Shen Q Wang R Guo Y Zhang C. Comparative analysis of the bioactive compounds in chicken cartilage: protective effects of chondroitin sulfate and type II collagen peptides against osteoarthritis involve gut microbiota. Front Nutr. (2022) 9:843360. 10.3389/fnut.2022.843360 35433786 PMC9005812

[14] Oliviero F Ramonda R Hoxha A Scanu A Galozzi P Favero M. Effect of an oral preparation containing hyaluronic acid, chondroitin sulfate, hydrolyzed collagen type II and hydrolyzed keratin on synovial fluid features and clinical indices in knee osteoarthritis. A pilot study. Reumatismo. (2020) 72:125–130. 10.4081/reumatismo.2020.1272 33213125

[15] Winther K Apel K Thamsborg G. A powder made from seeds and shells of a rose-hip subspecies (rosa canina) reduces symptoms of knee and hip osteoarthritis: a randomized, double-blind, placebo-controlled clinical trial. Scand J Rheumatol. (2005) 34:302–308. 10.1080/03009740510018624 16195164

[16] Ginnerup-Nielsen E Christensen R Bliddal H Zangger G Hansen L Henriksen M. Improved gait in persons with knee related mobility limitations by a rosehip food supplement: a randomized, double-blind, placebo-controlled trial. Gait Posture. (2015) 42:340–347. 10.1016/j.gaitpost.2015.07.001 26234471

[17] Schwager J Richard N Schoop R Wolfram S. A novel rose hip preparation with enhanced anti-inflammatory and chondroprotective effects. Mediators Inflamm. (2014) 2014:105710–10. 10.1155/2014/105710 25371599 PMC4211164

[18] Feistel B Walbroel B. A purified rose hip extract—effective in a new joint health concept. Z Für Phytother. (2012) 33. 10.1055/s-0032-1313250

[19] Le Bourgot C Rigaudier F Herpin F Juhel C. Randomised evaluation of gastrointestinal tolerance to high daily intake of short chain fructo-oligosaccharides in school-aged children. Nutrition. (2026) 113386:113386. 10.1016/j.nut.2026.113386 42710411

[20] Guy M Juhel C Rigaudier F Foucher C Mayeux G Sichère P. Mise en évidence d’une épargne thérapeutique antalgique grâce à l’utilisation de la neurostimulation électrique transcutanée pour le soulagement des douleurs articulaires et musculosquelettiques chroniques: essai contrôlé, randomisé, conduit en ouvert et en cross-over. Douleurs Éval - Diagn - Trait. (2021) 22:175–186. 10.1016/j.douler.2021.05.004

[21] Schön C Knaub K Alt W Durkee S Saiyed Z Juturu V. UC-II undenatured type II collagen for knee joint flexibility: a multicenter, randomized, double-blind, placebo-controlled clinical study. J Integr Complement Med. (2022) 28:540–548. 10.1089/jicm.2021.0365 PMC9232232 35377244

[22] Roos EM Roos HP Lohmander LS Ekdahl C Beynnon BD. Knee injury and osteoarthritis outcome score (KOOS)–development of a self-administered outcome measure. J Orthop Sports Phys Ther. (1998) 28:88–96. 10.2519/jospt.1998.28.2.88 9699158

[23] Ornetti P Parratte S Gossec L Tavernier C Argenson J-N Roos EM. Cross-cultural adaptation and validation of the French version of the knee injury and osteoarthritis outcome score (KOOS) in knee osteoarthritis patients. Osteoarthritis Cartilage. (2008) 16:423–428. 10.1016/j.joca.2007.08.007 17905602

[24] Ware J Kosinski M Keller SD. A 12-item short-form health survey: construction of scales and preliminary tests of reliability and validity. Med Care. (1996) 34:220–233. 10.1097/00005650-199603000-00003 8628042

[25] Moreton BJ Wheeler M Walsh DA Lincoln NB. Rasch analysis of the intermittent and constant osteoarthritis pain (ICOAP) scale. Osteoarthritis Cartilage. (2012) 20:1109–1115. 10.1016/j.joca.2012.06.011 22771772 PMC3526788

[26] Bolognese JA Schnitzer TJ Ehrich EW. Response relationship of VAS and Likert scales in osteoarthritis efficacy measurement. Osteoarthritis Cartilage. (2003) 11:499–507. 10.1016/S1063-4584(03)00082-7 12814613

[27] Kumavat R Kumar V Malhotra R Pandit H Jones E Ponchel F. Biomarkers of joint damage in osteoarthritis: current status and future directions. Mediators Inflamm. (2021) 2021:5574582. 10.1155/2021/5574582 33776572 PMC7969115

[28] Harris KK Dawson J Jones LD Beard DJ Price AJ. Extending the use of PROMs in the NHS—using the Oxford knee score in patients undergoing non-operative management for knee osteoarthritis: a validation study. BMJ Open. (2013) 3:e003365. 10.1136/bmjopen-2013-003365 23965934 PMC3753511

[29] Silva MDC Perriman DM Fearon AM Couldrick JM Scarvell JM. Minimal important change and difference for knee osteoarthritis outcome measurement tools after non-surgical interventions: a systematic review. BMJ Open. (2023) 13:e063026. 10.1136/bmjopen-2022-063026 37202126 PMC10201231

[30] Boonstra AM Schiphorst Preuper HR Balk GA Stewart RE. Cut-off points for mild, moderate, and severe pain on the visual analogue scale for pain in patients with chronic musculoskeletal pain. Pain. (2014) 155:2545–2550. 10.1016/j.pain.2014.09.014 25239073

[31] Kviatkovsky SA Hickner RC Cabre HE Small SD Ormsbee MJ. Collagen peptides supplementation improves function, pain, and physical and mental outcomes in active adults. J Int Soc Sports Nutr. (2023) 20:2243252. 10.1080/15502783.2023.2243252 37551682 PMC10411303

[32] Salinas-Camargo JC Marín-Pagán C Álvarez-Gil RV Martínez-Noguera FJ Cabrera-Cabrera M Llabrés-Laguarda JM. Effects of a food supplement containing hydrolyzed collagen on pain perception, joint range, and quality of life in people with chronic knee pain. Diseases. (2025) 13:189. 10.3390/diseases13070189 40709979 PMC12293524

[33] Khatri M Naughton RJ Clifford T Harper LD Corr L. The effects of collagen peptide supplementation on body composition, collagen synthesis, and recovery from joint injury and exercise: a systematic review. Amino Acids. (2021) 53:1493–1506. 10.1007/s00726-021-03072-x 34491424 PMC8521576

[34] Galluccio F Barskova T Cerinic MM. Short-term effect of the combination of hyaluronic acid, chondroitin sulfate, and keratin matrix on early symptomatic knee osteoarthritis. Eur J Rheumatol. (2015) 2:106–108. 10.5152/eurjrheum.2015.0019 27708941 PMC5047250

[35] Fladerer-Grollitsch J-P Klein T Kompek A Steiner N Menzel D Schön C. Effects of cartilage-supporting nutritional supplementation on knee osteoarthritis symptoms and quality of life in a 12-week randomized double-blind placebo-controlled pilot study. Sci Rep. (2025) 15:25625. 10.1038/s41598-025-11723-2 40664872 PMC12264193

[36] Schauss AG Stenehjem J Park J Endres JR Clewell A. Effect of the novel low molecular weight hydrolyzed chicken sternal cartilage extract, BioCell collagen, on improving osteoarthritis-related symptoms: a randomized, double-blind, placebo-controlled trial. J Agric Food Chem. (2012) 60:4096–4101. 10.1021/jf205295u 22486722

[37] Chen C-C Chang S-S Chang C-H Hu C-C Nakao Y Yong SM. Randomized, double-blind, four-arm pilot study on the effects of chicken essence and type II collagen hydrolysate on joint, bone, and muscle functions. Nutr J. (2023) 22:17. 10.1186/s12937-023-00837-w 36918892 PMC10015953

[38] Dubey V Kheni D Sureja V. Efficacy evaluation of standardized boswellia serrata extract (aflapinⓇ) in osteoarthritis: a systematic review and sub-group meta-analysis study. Explore. (2024) 20:102983. 10.1016/j.explore.2024.02.001 38365549

[39] Sengupta K Krishnaraju AV Vishal AA Mishra A Trimurtulu G Sarma KVS. Comparative efficacy and tolerability of 5-loxin and aflapin against osteoarthritis of the knee: a double blind, randomized, placebo controlled clinical study. Int J Med Sci. (2010) 7:366–377. 10.7150/ijms.7.366 21060724 PMC2974165

[40] Rao PS Ramanjaneyulu YS Prisk VR Schurgers LJ. A combination of tamarindus indica seeds and curcuma longa rhizome extracts improves knee joint function and alleviates pain in non-arthritic adults following physical activity. Int J Med Sci. (2019) 16:845–853. 10.7150/ijms.32505 31337958 PMC6643110

[41] Warholm O Skaar S Hedman E Mølmen HM Eik L. The effects of a standardized herbal remedy made from a subtype of Rosa canina in patients with osteoarthritis: a double-blind, randomized, placebo-controlled clinical trial. Curr Ther Res Clin Exp. (2003) 64:21–31. 10.1016/S0011-393X(03)00004-3 24944354 PMC4053021

[42] Rein E Kharazmi A Winther K. A herbal remedy, hyben vital (stand. Powder of a subspecies of Rosa canina fruits), reduces pain and improves general wellbeing in patients with osteoarthritis–a double-blind, placebo-controlled, randomised trial. Phytomedicine Int J Phytother Phytopharm. (2004) 11:383–391. 10.1016/j.phymed.2004.01.001 15330493

[43] Winther K. Shells from rose-hip (rosa canina) do not reduce symptom scores or improve anti-inflammatory property in patients with osteoarthritis—a double-blind, placebo-controlled, randomized study. Osteoarthritis Cartilage. (2014) 22(S321):S321. 10.1016/j.joca.2014.02.594

[44] Christensen R Tarp S Altman RD Henriksen M Bartels EM Klokker L. Comparing different preparations and doses of rosehip powder in patients with osteoarthritis of the knee: an exploratory randomized active-controlled trial. Int J Clin Rheumatol. (2014) 9:267–278. 10.2217/ijr.14.13

[45] Walbroel B Feistel B. A new refined rose hip extract and its anti-arthritic potential. Z Für Phytother. (2009) 30. 10.1055/s-0029-1239932

[46] Bi X. Correlation of serum cartilage oligomeric matrix protein with knee osteoarthritis diagnosis: a meta-analysis. J Orthop Surg. (2018) 13:262. 10.1186/s13018-018-0959-y PMC6195708 30340615

[47] Lambova SN Batsalova T Moten D Dzhambazov B. Cartilage oligomeric matrix protein in osteoarthritis and obesity—do new considerations emerge? Int J Mol Sci. (2024) 25:5263. 10.3390/ijms25105263 38791302 PMC11121003

[48] Happonen KE Saxne T Aspberg A Mörgelin M Heinegård D Blom AM. Regulation of complement by COMP allows for a novel molecular diagnostic principle in rheumatoid arthritis. Arthritis Rheum. (2010) 62:3574–3583. 10.1002/art.27720 20737467 PMC4576017

[49] Huang C Guo Z Feng Z Xu J Pan Z Liu W. Comparative study on the association between types of physical activity, physical activity levels, and the incidence of osteoarthritis in adults: the NHANES 2007–2020. Sci Rep. (2024) 14:20574. 10.1038/s41598-024-71766-9 39232062 PMC11374984

[50] Azukizawa M Ito H Hamamoto Y Fujii T Morita Y Okahata A. The effects of well-rounded exercise program on systemic biomarkers related to cartilage metabolism. Cartilage. (2019) 10:451–458. 10.1177/1947603518767998 29644876 PMC6755879

[51] Hay AM Rhoades MJ Bangerter S Ferguson SA Lee H T. Gill M. Serum cartilage oligomeric matrix protein concentration increases more after running than swimming for older people. Sports Health. (2024) 16:534–541. 10.1177/19417381231195309 37697665 PMC11195858

[52] Mündermann A Dyrby CO Andriacchi TP King KB. Serum concentration of cartilage oligomeric matrix protein (COMP) is sensitive to physiological cyclic loading in healthy adults. Osteoarthritis Cartilage. (2005) 13:34–38. 10.1016/j.joca.2004.09.007 15639635

[53] Hyldahl RD Evans A Kwon S Ridge ST Robinson E Hopkins JT. Running decreases knee intra-articular cytokine and cartilage oligomeric matrix concentrations: a pilot study. Eur J Appl Physiol. (2016) 116:2305–2314. 10.1007/s00421-016-3474-z 27699484

[54] Benito MJ Veale DJ FitzGerald O Van Den Berg WB Bresnihan B. Synovial tissue inflammation in early and late osteoarthritis. Ann Rheum Dis. (2005) 64:1263–1267. 10.1136/ard.2004.025270 15731292 PMC1755629

[55] Sokolove J Lepus CM. Role of inflammation in the pathogenesis of osteoarthritis: latest findings and interpretations. Ther Adv Musculoskelet Dis. (2013) 5:77–94. 10.1177/1759720X12467868 23641259 PMC3638313

[56] Haywood L McWilliams DF Pearson CI Gill SE Ganesan A Wilson D. Inflammation and angiogenesis in osteoarthritis. Arthritis Rheum. (2003) 48:2173–2177. 10.1002/art.11094 12905470

[57] Dwivedi G Flaman L Alaybeyoglu B Struglics A Frank EH Chubinskya S. Inflammatory cytokines and mechanical injury induce post-traumatic osteoarthritis-like changes in a human cartilage-bone-synovium microphysiological system. Arthritis Res Ther. (2022) 24:198. 10.1186/s13075-022-02881-z 35982461 PMC9386988

[58] Borst JM Ruoss S Palmer I Smith T Kalunian K Ward SR. Placebo effect sizes in clinical trials of knee osteoarthritis using intra-articular injections of biologic agents. Arthritis Care Res. (2025) 77:998–1006. 10.1002/acr.25526 PMC12307098 40105673

[59] Atasoy-Zeybek A Showel KK Nagelli CV Westendorf JJ Evans CH. The intersection of aging and estrogen in osteoarthritis. Npj Womens Health. (2025) 3:15. 10.1038/s44294-025-00063-1 40017990 PMC11860234

[60] Chodkowski J Grabowski F Rulewska N. The influence of physical activity on the development of osteoarthritis: a review. Qual Sport. (2024) 34:56206–56206. 10.12775/QS.2024.34.56206

[61] Zampogna B Papalia R Papalia GF Campi S Vasta S Vorini F. The role of physical activity as conservative treatment for hip and knee osteoarthritis in older people: a systematic review and meta-analysis. J Clin Med. (2020) 9:1167. 10.3390/jcm9041167 32325775 PMC7230847

