# Supplementary material for: Supplementation with cartilage hydrolysate and rosehip peel extract in physically active adults: main outcomes and exploratory findings of a randomized, placebo-controlled study on knee discomfort
Source: Front Sports Act Living. 2026 Oct 23;8:1873793. doi: 10.3389/fspor.2026.1873793 (PMC13741622; doi:10.3389/fspor.2026.1873793)
Supplement: Supplementary file 1 [file Table1.docx]

**Table S1.** Pain and functional scores at baseline per study arm and subgroup in the FAS.

|  | **Placebo** | | | | **CM** | | | |
| --- | --- | --- | --- | --- | --- | --- | --- | --- |
| **Variable** | All (n=68) | Men (n=26) | PreMW (n=28) | PostMW (n=14) | All (n=70) | Men (n=29) | PreMW (n=26) | PostMW (n=15) |
| **SLSD-related pain (0-10)** |  |  |  |  |  |  |  |  |
| Mean (SD) | 5.8 (0.8) | 5.6 (0.6) | 5.8 (0.8) | 6.2 (1.0) | 5.9 (0.8) | 5.9 (0.7) | 6.0 (1.0) | 5.6 (0.7) |
| Median (Q1; Q3) | 5.6 (5.2; 6.1) | 5.6 (5.2; 5.9) | 5.6 (5.2; 6.3) | 5.9 (5.3; 7.1) | 5.6 (5.3; 6.2) | 5.8 (5.4; 6.2) | 5.5 (5.2; 6.5) | 5.5 (5.1; 5.7) |
| **Knee range of motion** |  |  |  |  |  |  |  |  |
| - Active flexion (degree) |  |  |  |  |  |  |  |  |
| Mean (SD) | 130.7 (8.3) | 129.5 (8.8) | 133.3 (7.4) | 127.8 (8.0) | 130.9 (7.2) | 130.6 (8.2) | 131.9 (6.5) | 129.7 (6.3) |
| Median (Q1; Q3) | 131.0 (123.0; 136.5) | 131.0 (120.0; 136.0) | 132.0 (130.0; 138.0) | 127.0 (120.0; 136.0) | 130.0 (128.0; 135.0) | 130.0 (125.0; 136.0) | 130.0 (130.0; 135.0) | 130.0 (125.0; 132.0) |
| - Extension (degree) |  |  |  |  |  |  |  |  |
| Mean (SD) | 2.1 (3.1) | 2.2 (3.6) | 2.4 (3.1) | 1.5 (2.0) | 2.5 (3.2) | 3.0 (3.6) | 2.4 (3.0) | 1.9 (2.7) |
| Median (Q1; Q3) | 0.0 (0.0; 4.5) | 0.0 (0.0; 5.0) | 0.0 (0.0; 5.0) | 0.5 (0.0; 2.0) | 0.0 (0.0; 5.0) | 2.0 (0.0; 5.0) | 0.0 (0.0; 5.0) | 0.0 (0.0; 5.0) |
| **ICOAP scores (arbitrary unit)** |  |  |  |  |  |  |  |  |
| - Constant pain |  |  |  |  |  |  |  |  |
| Mean (SD) | 26.8 (15.7) | 25.0 (15.4) | 25.5 (14.4) | 32.5 (18.6) | 26.2 (15.0) | 26.9 (13.7) | 26.5 (17.6) | 24.3 (13.6) |
| Median (Q1; Q3) | 25.0 (15.0; 35.0) | 25.0 (15.0; 35.0) | 25.0 (15.0; 35.0) | 30.0 (20.0; 40.0) | 22.5 (15.0; 35.0) | 25.0 (20.0; 30.0) | 20.0 (15.0; 35.0) | 20.0 (10.0; 35.0) |
| - Intermittent pain |  |  |  |  |  |  |  |  |
| Mean (SD) | 32.4 (15.5) | 29.5 (15.7) | 31.8 (14.1) | 39.0 (16.8) | 34.0 (15.3) | 34.6 (15.9) | 34.54 (15.7) | 32.2 (14.3) |
| Median (Q1; Q3) | 29.2 (20.8; 39.6) | 29.2 (16.7; 37.5) | 29.2 (22.9; 37.5) | 37.5 (29.2; 45.8) | 31.3 (20.8; 45.8) | 33.3 (25.0; 41.7) | 29.2 (20.8; 50.0) | 29.2 (20.8; 45.8) |
| **KOOS scores (arbitrary unit)** |  |  |  |  |  |  |  |  |
| - Global |  |  |  |  |  |  |  |  |
| Mean (SD) | 66.8 (12.1) | 66.5 (11.0) | 67.0 (12.0) | 66.8 (15.0) | 66.8 (11.8) | 64.4 (10.4) | 68.7 (11.5) | 68.0 (14.5) |
| Median (Q1; Q3) | 68.4 (56.9; 76.2) | 68.8 (58.4; 76.7) | 66.9 (55.9; 75.6) | 69.9 (59.5; 76.1) | 65.8 (57.6; 76.5) | 60.8 (56.6; 71.5) | 67.1 (59.8; 75.8) | 70.6 (55.9; 82.9) |
| - Pain |  |  |  |  |  |  |  |  |
| Mean (SD) | 68.1 (12.3) | 67.3 (11.0) | 69.3 (12.7) | 67.1 (14.2) | 68.9 (11.2) | 67.8 (10.6) | 69.7 (11.9) | 69.8 (11.7) |
| Median (Q1; Q3) | 69.4 (58.3; 77.8) | 68.1 (58.3; 77.8) | 70.8 (58.3; 79.2) | 69.4 (63.9; 75.0) | 65.8 (57.6; 76.5) | 69.4 (61.1; 75.0) | 70.8 (61.1; 77.8) | 72.2 (63.9; 77.8) |
| - Other symptoms |  |  |  |  |  |  |  |  |
| Mean (SD) | 74.5 (12.1) | 73.2 (11.2) | 76.7 (11.9) | 72.4 (14.3) | 74.6 (11.5) | 73.4 (10.7) | 76.8 (10.5) | 73.1 (14.4) |
| Median (Q1; Q3) | 76.8 (66.1; 82.1) | 75.0 (64.3; 82.1) | 78.6 (67.9; 85.7) | 76.8 (67.9; 82.1) | 75.0 (64.3; 82.1) | 75.0 (64.3; 82.1) | 78.6 (71.4; 85.7) | 71.4 (64.3; 82.1) |
| - Activity of daily living |  |  |  |  |  |  |  |  |
| Mean (SD) | 79.2 (14.8) | 80.5 (13.2) | 79.8 (14.2) | 75.5 (18.8) | 77.3 (12.5) | 73.7 (12.5) | 81.5 (11.6) | 76.9 (12.6) |
| Median (Q1; Q3) | 83.8 (69.9; 90.4) | 83.8 (77.9; 89.7) | 83.8 (64.7; 91.9) | 83.8 (69.1; 88.2) | 75.0 (64.3; 82.1) | 76.5 (63.2; 82.4) | 84.6 (73.5; 91.2) | 80.9 (69.1 (88.2) |
| - Sport and recreation |  |  |  |  |  |  |  |  |
| Mean (SD) | 52.9 (18.6) | 53.5 (16.2) | 51.1 (20.1) | 55.4 (20.8) | 51.8 (18.7) | 48.8 (19.3) | 54.2 (16.9) | 55.3 (20.8) |
| Median (Q1; Q3) | 52.5 (40.0; 65.0) | 55.0 (40.0; 65.0) | 45.0 (40.0; 62.5) | 52.5 (40.0; 65.0) | 50.0 (40.0; 65.0) | 45.0 (40.0; 60.0) | 50.0 (40.0; 65.0) | 55.0 (30.0; 75.0) |
| - Knee-related quality of life |  |  |  |  |  |  |  |  |
| Mean (SD) | 59.3 (14.9) | 57.9 (13.2) | 58.3 (14.8) | 63.8 (17.9) | 61.4 (17.2) | 58.4 (15.8) | 61.5 (16.2) | 67.1 (20.8) |
| Median (Q1; Q3) | 62.5 (50.0; 68.8) | 59.4 (50.0; 68.8) | 59.4 (46.9; 68.8) | 68.8 (50.0; 75.0) | 62.5 (50.0; 75.0) | 56.3 (50.0; 68.8) | 62.5 (50.0; 75.0) | 75.0 (50.0; 81.3) |

*CM: Chondractiv™ Move. PreMW: premenopausal women. PostMW: postmenopausal women. SD: standard deviation; Q1 / Q3: 1^st^ / 3^rd^ quartile. KOOS: Knee injury and Osteoarthritis Outcome Score. ICOAP:Intermittent and Constant Osteoarthritis Pain; SLSD: single-leg step-down test*
