# Supplementary material for: Supplementation with cartilage hydrolysate and rosehip peel extract in physically active adults: main outcomes and exploratory findings of a randomized, placebo-controlled study on knee discomfort
Source: Front Sports Act Living. 2026 Oct 23;8:1873793. doi: 10.3389/fspor.2026.1873793 (PMC13741622; doi:10.3389/fspor.2026.1873793)
Supplement: Supplementary file 2 [file Table2.docx]

**Table S2.** Quality of life scores and biomarker levels at baseline per study arm and subgroup in the FAS.

|  | **Placebo** | | | | **CM** | | | |
| --- | --- | --- | --- | --- | --- | --- | --- | --- |
| **Variable** | All (n=68) | Men (n=26) | PreMW (n=28) | PostMW (n=14) | All (n=70) | Men (n=29) | PreMW (n=26) | PostMW (n=15) |
| **SF-12 (arbitrary unit)** |  |  |  |  |  |  |  |  |
| - Physical component score |  |  |  |  |  |  |  |  |
| Mean (SD) | 46.0 (7.7) | 47.6 (6.1) | 44.5 (8.6) | 46.1 (8.4) | 44.6 (7.0) | 43.4 (6.7) | 45.5 (7.4) | 45.5 (7.0) |
| Median (Q1; Q3) | 46.4 (39.3; 51.5) | 49.2 (44.0; 51.1) | 42.1 (38.2; 50.9) | 46.8 (36.3; 52.8) | 46.2 (38.4; 49.7) | 44.3 (37.9; 48.6) | 47.4 (39.4; 52.1) | 47.6 (38.2; 52.0) |
| - Mental component score |  |  |  |  |  |  |  |  |
| Mean (SD) | 48.5 (7.5) | 48.7 (6.7) | 47.6 (7.8) | 49.9 (8.8) | 49.9 (8.0) | 50.3 (6.6) | 49.5 (7.9) | 49.9 (10.8) |
| Median (Q1; Q3) | 49.8 (44.0; 53.8) | 49.2 (42.6; 53.5) | 48.9 (44.0; 52.2) | 51.5 (47.8; 58.8) | 52.3 (45.9; 55.9) | 51.1 (46.0; 54.4) | 51.9 (43.2; 56.1) | 53.7 (47.4; 55.6) |
| **Biomarkers (ng/mL)** |  |  |  |  |  |  |  |  |
| - COMP |  |  |  |  |  |  |  |  |
| Mean (SD) | 69.4 (37.1) | 67.3 (32.7) | 67.7 (31.5) | 76.5 (53.9) | 56.2 (18.5) | 54.9 (19.2) | 56.0 (18.0) | 59.1 (19.1) |
| Median (Q1; Q3) | 60.0 (45.5; 80.0) | 59.0 (46.0; 80.0) | 61.0 (45.5; 89.5) | 64.0 (45.0; 76.0) | 54.0 (44.0; 68.0) | 50.0 (42.0; 64.0) | 53.5 (46.0; 60.0) | 62.0 (47.0; 74.0) |
| - PIIANP |  |  |  |  |  |  |  |  |
| Mean (SD) | 162.0 (127.4) | 159.1 (118.5) | 160.4 (146.8) | 170.7 (108.4) | 154.4 (214.6) | 174.2 (145.4) | 179.7 (310.1) | 72.3 (62.2) |
| Median (Q1; Q3) | 110.5 (76.0; 234.5) | 110.5 (76.0; 237.0) | 103.0 (62.0; 240.0) | 135.5 (91.0; 232.0) | 93.5 (52.0; 193.0) | 93.5 (52.0; 193.0) | 99.0 (65.0; 193.0) | 52.0 (18.0; 91.0) |

*CM: Chondractiv™ Move. PreMW: premenopausal women. PostMW: postmenopausal women. SD: standard deviation; Q1 / Q3: 1^st^ / 3^rd^ quartile. SF-12: quality of life score; COMP: cartilage oligomeric matrix protein; PIIANP: type IIA collagen N-propeptide*
